# Development and evaluation of a novel system for inducing orthostatic challenge by tilt tests and lower body negative pressure

**DOI:** 10.1038/s41598-018-26173-2

**Published:** 2018-05-17

**Authors:** Łukasz Dziuda, Mariusz Krej, Maciej Śmietanowski, Aleksander Sobotnicki, Mariusz Sobiech, Piotr Kwaśny, Anna Brzozowska, Paulina Baran, Krzysztof Kowalczuk, Franciszek W. Skibniewski

**Affiliations:** 1 Department of Flight Simulator Innovations, Military Institute of Aviation Medicine, ul. Krasińskiego 54/56, 01-755 Warszawa, Poland; 2 Department of Experimental and Clinical Physiology, Medical University of Warsaw, ul. Banacha 1B, 02-097 Warszawa, Poland; 3 Department of Research and Development, Institute of Medical Technology and Equipment, ul. Roosevelta 118, 41-800, Zabrze, 41-800 Poland; 4 ETC-PZL Aerospace Industries Sp. z o.o., Aleja Krakowska 110/114, 02-256 Warszawa, Poland; 5 Department of Simulator Studies and Aeromedical Training, Military Institute of Aviation Medicine, ul. Krasińskiego 54/56, 01-755 Warszawa, Poland

## Abstract

Lower body negative pressure (LBNP) is a method derived from space medicine, which in recent years has been increasingly used by clinicians to assess the efficiency of the cardiovascular regulatory mechanisms. LBNP with combined tilt testing is considered as an effective form of training to prevent orthostatic intolerance. We have developed a prototype system comprising a tilt table and LBNP chamber, and tested it in the context of the feasibility of the device for assessing the pilots’ efficiency. The table allows for controlled tilting in the range from −45 to +80° at the maximum change rate of 45°/s. The LBNP value can smoothly be adjusted down to −100 mmHg at up to 20 mmHg/s. 17 subjects took part in the pilot study. A 24-minute scenario included −100 mmHg supine LBNP, head up tilt (HUT) and −60 mmHg LBNP associated with HUT, separated by resting phases. The most noticeable changes were observed in stroke volume (SV). During supine LBNP, HUT and the combined stimulus, a decrease of the SV value by 20%, 40% and below 50%, respectively, were detected. The proposed system can map any pre-programed tilt and LBNP profiles, and the pilot study confirmed the efficiency of performing experimental procedures.

## Introduction

A number of cardiovascular disorders are observed when human body is subjected to accelerations other than the gravity of Earth. Although the first consideration on the medical aspects of the influence of the variable gravitational field date back to 1818^1^, only the rapid development of human centrifuges, tilt tables and under- or overpressure chambers in the 1960s allowed for controlling the gravitational stimuli quantitatively^2–5^. Accelerations directed from head to feet (+G_z_) and accelerations acting oppositely (−G_z_) have been of particular interest to space and aviation medicine. In the first case, blood moves toward the lower extremities and its drain from the brain may cause oxygen deficiency leading in sequence to greyout, tunnel vision, blackout and G-induced loss of consciousness (G-LOC)^6^. The adverse symptoms of +G_z_ acceleration are additionally intensified if the positive stimulus is preceded by −G_z_ acceleration, i.e. a push-pull effect occurs^7^. The literature explains this increased regulatory response through the significant contribution of sympathetic activity^8^. A list of states deviating from the Earthly gravity is complemented by weightlessness and microgravity, i.e. zero- and near zero-gravity states, respectively. These aspects are mainly researched in the context of space missions during which, promptly upon entering weightlessness, blood shifts toward the head^9^. The cardiovascular system adapts to the new conditions after a time^10^, however blood content in the lower part of the body remains unnaturally low and long-term stay in space impairs vestibulo-cardiovascular reflex^11^ and leads to, e.g. a change in heart shape and loss of heart muscle mass^12^. Returns from space missions are also aggressive for the body due to symptoms accompanying them, such as orthostatic intolerance, hypotension, dizziness and syncope^13^. For the above-mentioned reasons, methods for increasing the body tolerance to +G_z_ and −G_z_ accelerations and for counteracting the disadvantageous effects of weightlessness are of fundamental meaning in space and aviation medicine.

In general, three methods for real or simulated changing direction and value of the gravity field vector are used:

- centrifugation,
- head up tilt (HUT) and head down tilt (HDT) tests,
- lower body negative pressure (LBNP) and lower body positive pressure (LBPP) tests.

Modern human centrifuges enable for mapping acceleration profiles that reflect flight conditions with great accuracy, and the pilot is subjected to real accelerations. Although the centrifuge is recognised as the best flight simulator, high costs of its construction, operation and maintenance have resulted in searching for alternatives. One of them is a tilt table. This device of modest complexity offers a generation of stimuli in the range from −1 g (tilt of −90°, HDT) to 1 g (tilt of +90°, HUT) in the G_z_ axis. Despite this limitation, even weak stimuli below 1 g in the G_z_ axis (+1 G_z_, in short) trigger a series of reflex reactions whose monitoring allows for assessing the efficiency of the cardiovascular regulatory mechanisms. On the other hand, placing the lower body of the pilot (usually from the waist area down) in an underpressure chamber simulates cardiovascular effects corresponding to stimuli greater than +1 G_z_. Some authors report on supine LBNP at −50 mmHg (millimetre of mercury), which induced the hemodynamic response similar to that observed at an acceleration value of +2 G_z_^14^. To simulate +2 G_z_ heart rate (HR) effects in weightlessness, an increase in LBNP to −90 mmHg is required^15^. Other authors attempt to prove that the LBNP test can be a more effective and practical stimulus than centrifugation^16^.

The underpressure chamber is often placed on the tilt table in order to strengthen the +G_z_ stimulus by using LBNP and moving the pilot from supine to vertical position simultaneously. Moreover, this configuration allows for achieving the push-pull effect when the LBNP and/or HUT exposures are preceded by a HDT try. Similarly to the HDT test, the LBPP technique aims to reverse or prevent the effects of orthostatic hypotension. It is however much less commonly used, mainly due to problems met when designing one device purposed for generating positive and negative pressures.

In this paper, we summarise the modelling phases of the ORTHO-LBNP system, i.e. a prototype device consisting of the tilt table to generate orthostatic stress and the underpressure chamber to extort pooling of blood in the lower extremities, as during +Gz accelerations. We also present results of the pilot study, in which the main emphasis is put to identify physiological indicators that best correlate with the level of arousal and enable for classifying the reflex cardiac response due to the degree of the subject’s resistance to accelerations. Finally, we propose an indicator to estimate orthostatic tolerance in pilots, and discuss the prototype system with regard to other known tilt tables with combined LBNP chambers. The main goal of the paper is to demonstrate the system that can map any pre-programed tilt and LBNP profiles for the needs of screening for orthostatic tolerance and training to improve G-tolerance in pilots.

## Results

### Modelling the tilt table and LBNP chamber

Previously, an LBNP chamber integrated with a tilt table was constructed at the Military Institute of Aviation Medicine, Poland, and the preliminarily data were presented^17^. Although the intention of those authors was to implement the device into the selection process of candidates for military pilots, deeper investigations were suspended, mainly due to the use of fast aging components causing frequent failures. The previous experiences as well as a comprehensive literature review, briefly shown in the Discussion section, were elementary for designing the prototype of the ORTHO-LBNP system, in which durable elements and modern measurement modules have been utilised.

In order for the device to be used both for carrying out selection procedures and researching clinical aspects, the tilt table must meet two main requirements:

- range of tilt angles: −45° to +80°,
- rate of tilt changes: up to 45°/s.

**Figure 1:**
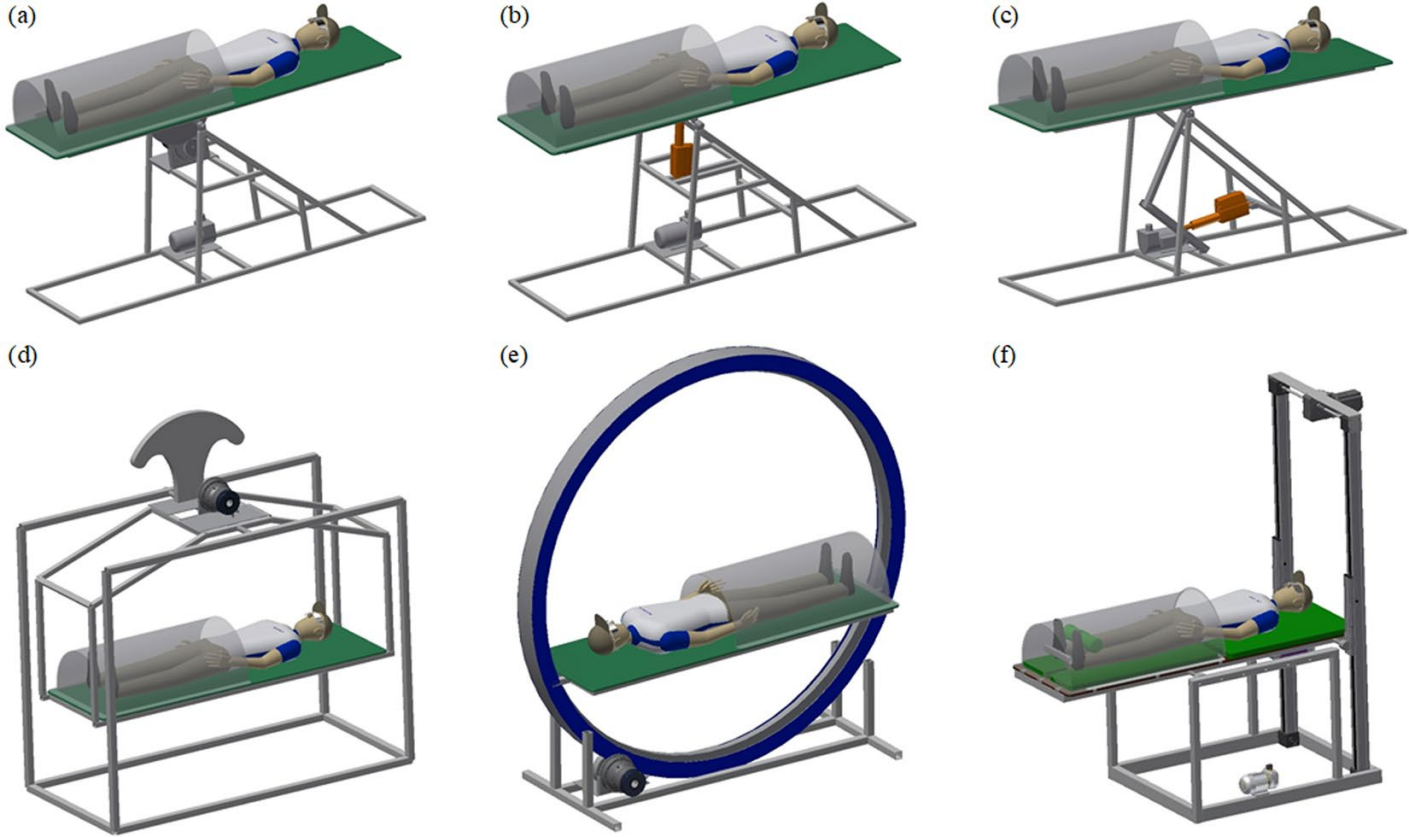
Considered models of the tilt table construction.

With regard to these requirements, the authors considered six models of the tilt table construction prepared in the *Autodesk Inventor* environment, shown in Fig. 1. Model 1 (Fig. 1(a)) is based on a rotary engine that directly transfers the change of the motor shaft angle to the change of the tilt table angle. These changes are proportional to each other. A linear actuator coupled to a fixed axis of rotation directly (Fig. 1(b)) or through an intermediate mechanism (Fig. 1(c)) is the drive module of model 2 and model 3, respectively. Linear motion of the actuator causes rotation of the tilt table. The stroke change of the actuator piston rod is not proportional to the change of the tilt table angle. In the three models mentioned above, the table movement is realised around the fixed axis of rotation, supported on a rigid frame of the device construction. The table tilt is similarly generated in a cradle-based model 4 (Fig. 1(d)), with the difference that the fixed axis of rotation is hanged above the table. A counterweight should be used to facilitate the movement. A completely different concept is model 5 whose principle of operation relies on placing the movable rim inside the fixed rim and driving the first one by a rotary motor (Fig. 1(e)). The tilt table is attached to the movable rim and it performs rotation around the virtual axis being centre of symmetry of the two rims. In model 6, the tilt table is placed on two axes terminated on both sides with guide blocks (Fig. 1(f)). One of the axes cooperates with a horizontal rail, whereas the other is associated with a vertical rail. The vertically moved guide blocks are driven by a rotary engine, and the horizontally moved guide blocks support the tilt table. In this case, the tilt table moves around a variable axis of rotation.

**Figure 2:**
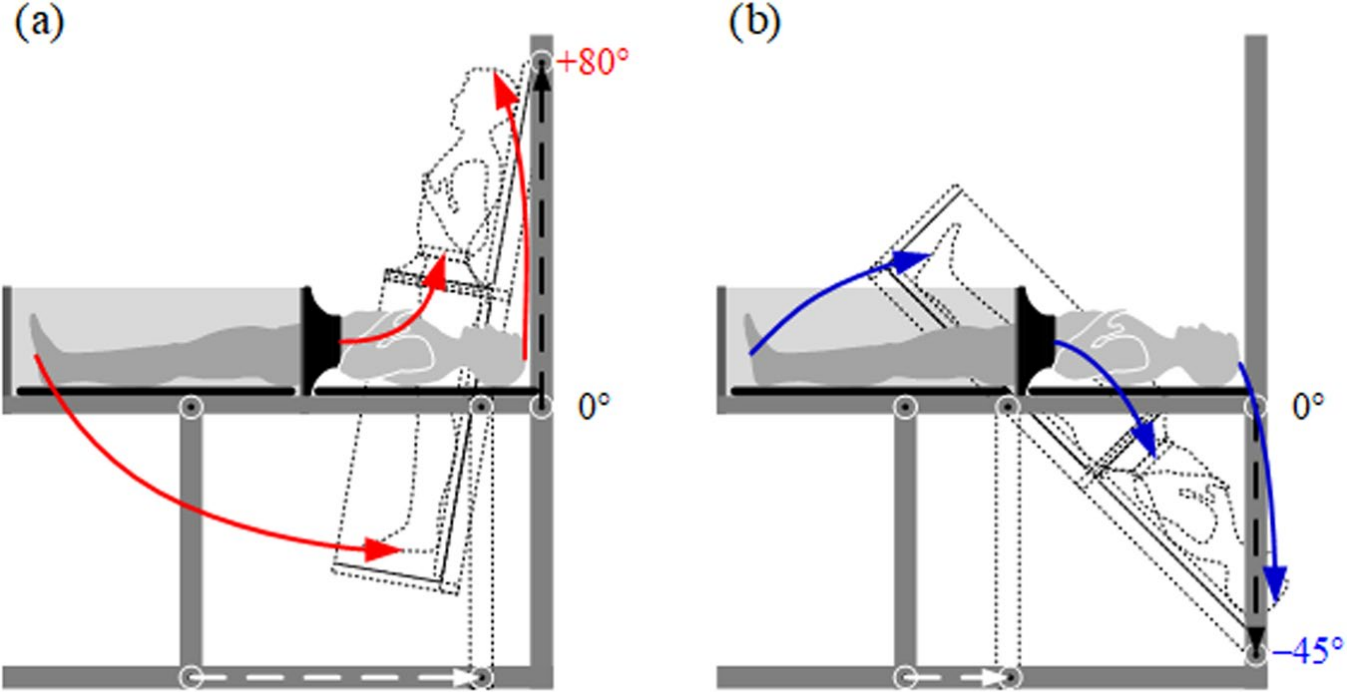
Trajectories of the body on the tilt table during (**a**) HUT and (**b**) HDT tests.

Among the proposed software models, one solution was chosen to build a physical model of the device. The selection was made by means of a criterion-based optimisation method including technical matters, safety considerations, ergonomics and economic aspects listed alphabetically in Table 1. Each of the sixteen criteria was evaluated by the engineering team using a 5-point scale, where 1 point was assigned when the model did not satisfy the criterion, and 5 points were allocated to the models excellently meeting the criterion. Although models 1–4 can be constructed at relatively low costs, they allow the operator to tilt the subject only around the fixed axis of rotation. The movement achieved in this way is far from an acceleration profile to which pilots are subjected in real flight. Model 5 offers more in this area. The subject is tilted around the virtual axis of rotation and can additionally be placed closer or away from this axis by adjusting the tilt table position within the inner rim. However, the device requires extremely precise fitting of the mechanical components, and thus higher outlay. The optimisation method indicated model 6 as the most optimal solution, in which the subject tilts around the variable axis of rotation. As shown in Fig. 2, the subject’s body slidingly moves in the head-to-feet axis while changing mainly the level of the upper part of the body, both in HUT and HDT tests. Trajectories of three human body characteristic points, i.e. head, waist and feet, during HUT and HDT tests are illustrated by the red and blue solid lines, respectively. Additionally, displacements of the guide blocks on the horizontal and vertical rails are marked with the white and black dotted lines, respectively. Such a movement of the subject lying on the tilt table can, to some extent, simulate flight conditions at a relatively low technical complexity and average costs. This drive subsystem has been registered in the Patent Office of the Republic of Poland^18^.

**Table 1:** Results of the tilt table construction optimisation.

Criterion	Model					
	**1**	**2**	**3**	**4**	**5**	**6**
Aesthetics	4	4	4	3	5	5
Areas dangerous to patient	3	3	3	2	4	3
Areas dangerous to staff	3	3	3	2	5	4
Complexity	4	4	3	2	2	4
Cost	4	4	4	4	1	3
Degree of precision	4	3	3	3	1	3
Dimensions	4	4	4	2	2	4
Ease of cleaning	4	4	3	3	2	3
Ease of leading cables	4	4	4	4	2	3
Easy to build	5	5	5	3	1	5
Ergonomics	2	2	2	2	3	3
Rigidity, durability	1	1	1	2	4	4
Threat in case of failure	2	2	2	3	4	3
Use of commercial elements	2	2	2	2	2	4
Variable rotation axis	1	1	1	1	3	5
Weight	4	4	4	3	2	4
Points total	51	50	48	41	43	60

Rating: 1 – poor, 2 – acceptable, 3 – good, 4 – very good, 5 – excellent.

To build the prototype device, the next step was to design a subsystem for generating underpressure including the LBNP chamber with its main requirements summarised as follows:

- range of generated underpressure: 0 to −100 mmHg,
- rate of underpressure changes: up to 20 mmHg/s.

Such values of negative pressure parameters in connection with the values of the tilt parameters, highlighted above, enable for building different shapes of underpressure and tilt profiles. This in turn allows for generating physiological disorders in the human body at a level sufficient to evaluate the subject’s resistance to accelerations or to develop his/her ability to adapt variable gravitational stimuli.

Initially, we considered the use of a buffer tank between the underpressure chamber and a compressor or vacuum pump to generate negative pressure. This required applying controllable valves between the buffer tank and the underpressure chamber, and a safety valve to reduce pressure in the tank. The compressor was to achieve a much greater negative pressure in the buffer tank than required in the underpressure chamber. In this way a low-flow compressor could be used to reduce pressure in the chamber at the required change rate. Nevertheless, the authors decided to engage only elements that meet the definition of low-pressure equipment according to Directive 2014/68/EU relating to the making available on the market of pressure equipment. Although this required the use of a high-flow compressor to achieve an appropriate pressure dynamics in the chamber, the subsystem for generating underpressure was simplified by eliminating the buffer tank. Additionally, the maximum negative pressure achieved by the compressor can be lower than that in the case of a compressor operating in the configuration with the buffer tank.

**Figure 3:**
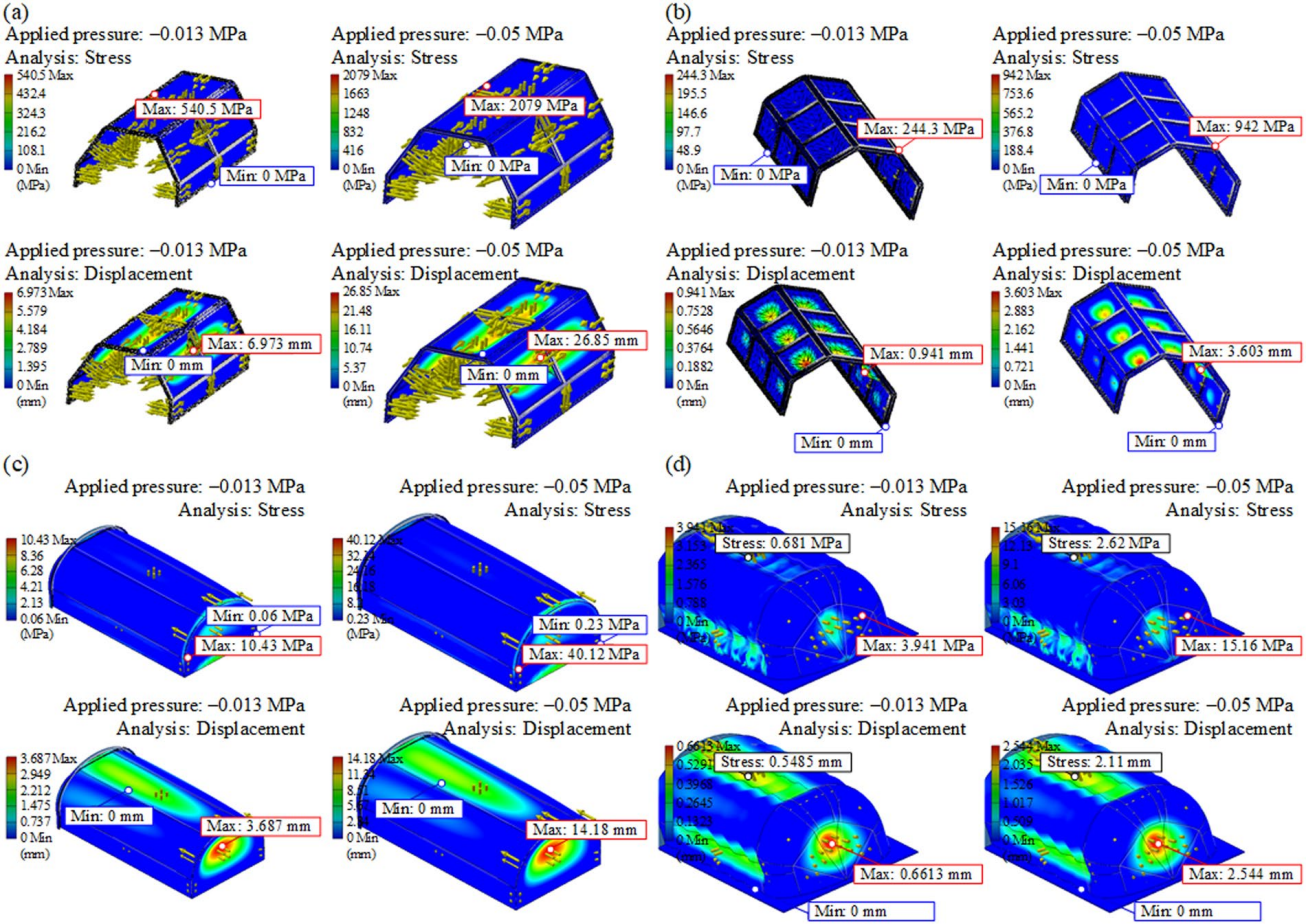
Strength analysis of the chamber based on an (**a**) Al frame, 1 transverse beam, 30° slope, PC inserts, 5 mm thick, (**b**) Al frame, 2 transverse beams, 45° slope, PC inserts, 6 mm thick, (**c**) thermoformed PC sheet, 8 mm thick and (**d**) thermoformed corrugated PC sheet, 6 mm thick.

We verified four variants of the chamber based on an aluminium (Al) frame with polycarbonate (PC) inserts (variant 1 and 2), thermoformed PC sheet (variant 3), which could be additionally corrugated (variant 4). The considered variants were subjected to a simulated pressure effect of −0.013 MPa, i.e. the required −100 mmHg. For safety reasons, the behaviour of particular variants of the chamber subjected to a pressure of −0.05 MPa (−375 mmHg) was also verified. As a result of numerical simulations, the maps of equivalent stresses and total displacements for particular segments of the chamber, shown in Fig. 3, were obtained. The Al frame-based chamber shown in Fig. 3(a) experiences significant stress under negative pressure and the PC inserts may be damaged. An additional transverse beam in the chamber frame allows to withstand the required underpressure without damage to the PC, as shown in Fig. 3(b). Alternatively, significantly better strength properties are achieved by the chamber made of thermally bent PC, shown in Fig. 3(c). The strength can be additionally improved by corrugating the surface of the PC (Fig. 3(d)), which, however, raises costs. The strength analysis and ease of construction indicated variant 3 as the most optimal one that was adopted for building the prototype of the ORTHO-LBNP system.

### Pilot study

The pilot study was conducted on a group of 17 healthy cadets of the Polish Air Force Academy including 3 women and 14 men; age: 24.1 ± 1.6 (mean ± SD) years, weight: 73.6 ± 14.0 kg, and height: 177.0 ± 8.5 cm.

The experimental protocol was composed of seven phases that can be characterised as: (1) 300-s check before the stimuli, (2) LBNP applied stepwise with 11.1 mmHg/15 s decrement to −100 mmHg, and then this value is sustained for 120 s, (3) 180-second phase of rest between stimuli, (4) 75°-HUT (5°/s) for 120 s after a 15-s reversing of the gravity vector (−30°), (5) 180-second phase of rest between stimuli, (6) 75°-HUT (5°/s) accompanied by an exposure to an LBNP of −60 mmHg increased linearly by −4 mmHg/s, and then this value is sustained for 120 s during HUT, and (7) 120-s check after the stimuli.

**Figure 4:**
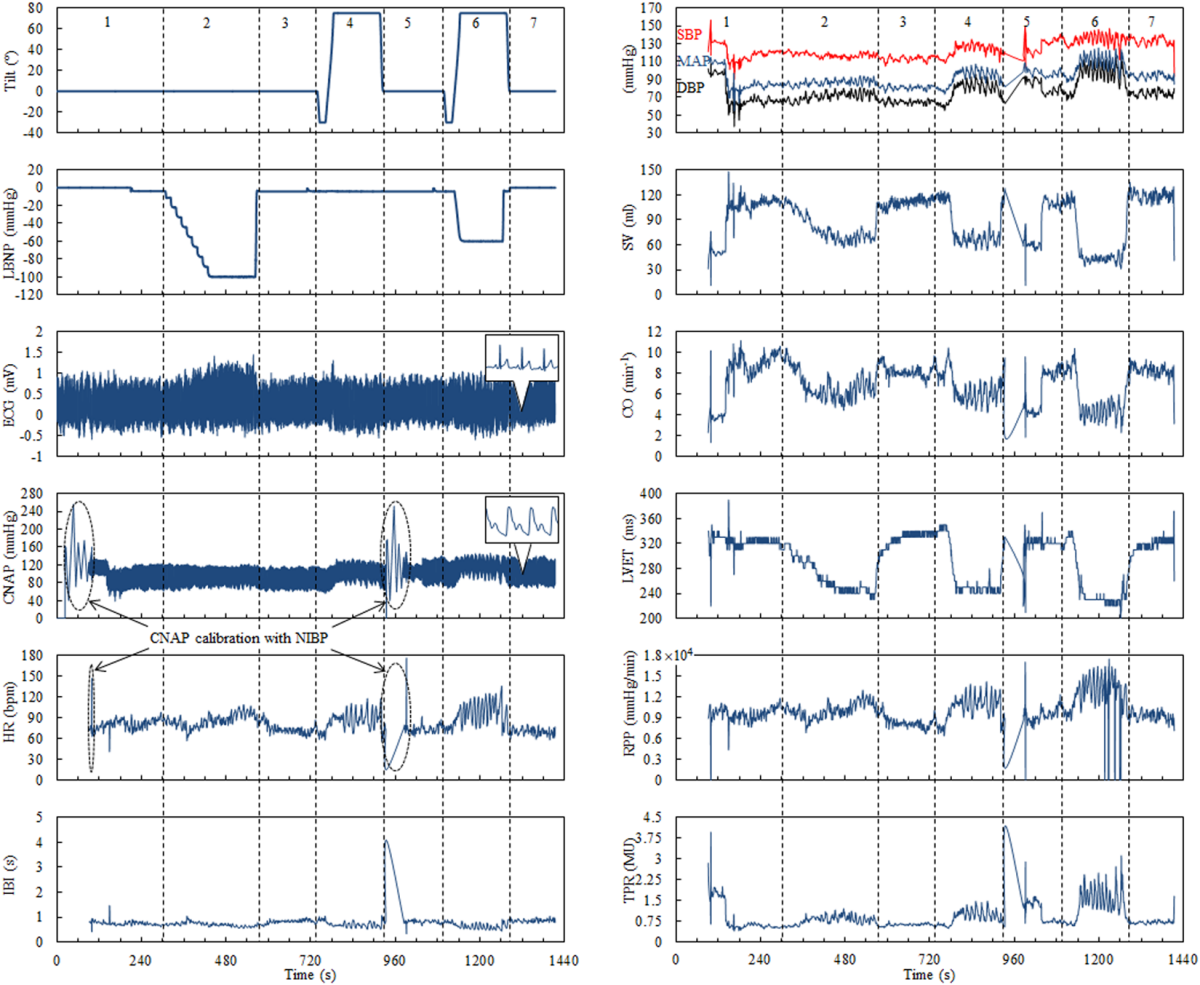
Example of an original recording: table tilt, LBNP profile, ECG signal, CNAP wave, and traces of the physiological parameters extracted from the ECG and CNAP signals in the subsequent phases of the pilot study.

Figure 4 shows the changing tilt of the table and negative pressure as well as the ECG signal and continuous non-invasive arterial pressure (CNAP) wave in one of the subjects (subject no. 10, man, 23 years old, weight: 70 kg, height: 177 cm) recorded over the 24-minute scenario. The vertical dotted lines show the boundaries between the individual experiment phases. At the beginning of phases 1 and 5, the CNAP module was automatically calibrated in relation to an upper-arm non-invasive blood pressure (NIBP) meter. Although this disturbed the CNAP measurement, it made it possible to obtain the most reliable blood pressure records and improved the subject’s comfort by the change of the finger on which the measurement was done using a miniature cuff. HR and inter-beat interval (IBI) were calculated using the ECG and CNAP signals. Systolic and diastolic blood pressure (SBP and DBP, respectively), mean arterial pressure (MAP), stroke volume (SV), cardiac output (CO), left ventricular ejection time (LVET), rate pressure product (RPP), and total peripheral resistance (TPR) were extracted from the CNAP wave. All of them are depicted in relation to the study phases in Fig. 4.

**Figure 5:**
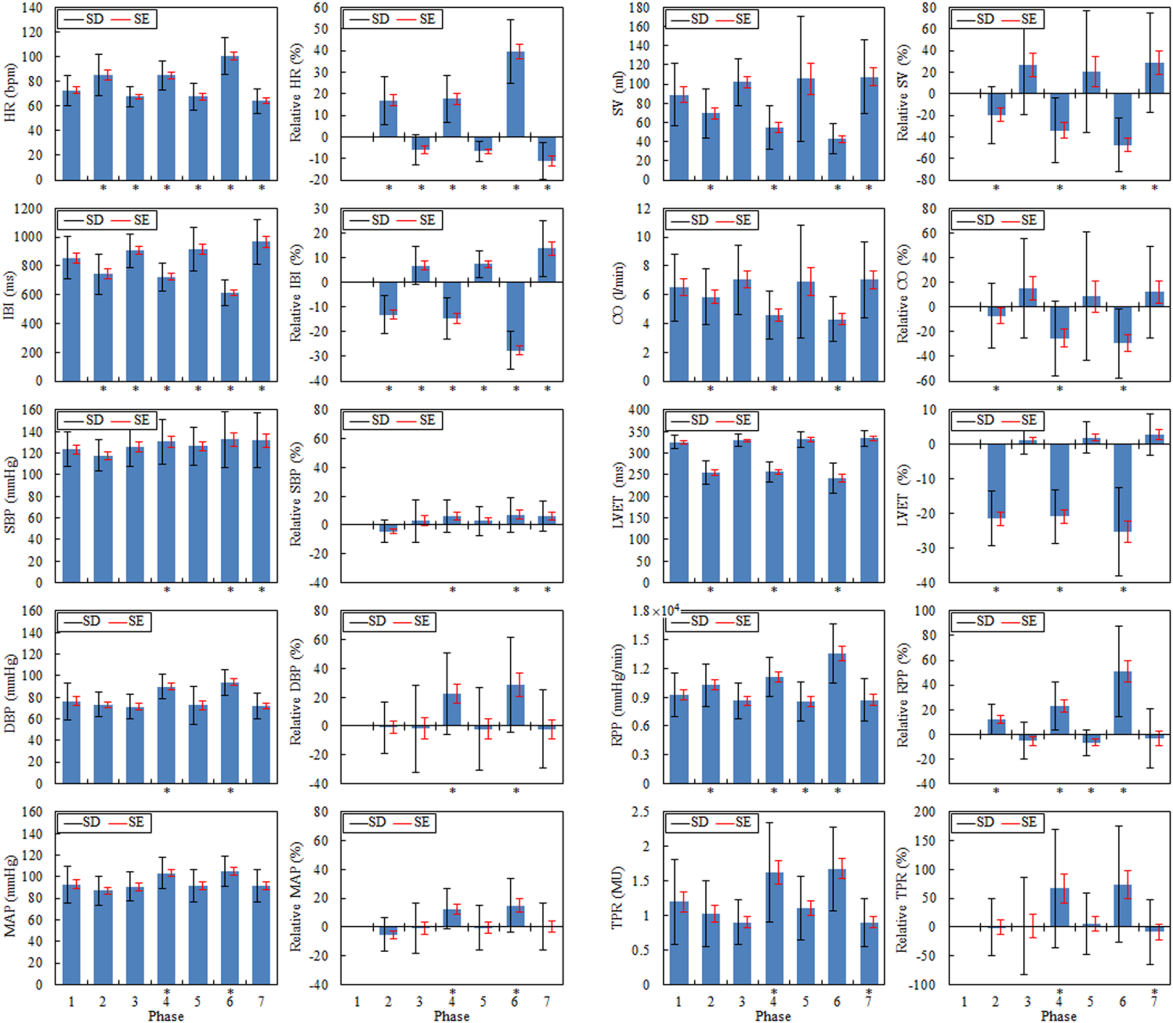
Absolute and relative changes in HR, IBI, SBP, DBP, MAP, SV, CO, LVET, RPP and TPR in the subsequent phases of the pilot study. Asterisks indicate the statistical significance (p ≤ 0.05).

This paper refers exclusively to results statistically reliable or on the borderline of statistical significance. They include the mean values of HR, IBI, SV, CO, LVET, RPP, and, to a limited extent, MAP and TPR. Figure 5 presents the absolute and relative (in relation to the initial level in phase 1) values of the means of the individual parameters with standard deviation (±SD) and standard error (±SE) in the separate study phases. In addition to the graphical presentation of data, Table 2 includes numerical data and significance levels for changes in the mean values obtained in the subsequent phases with respect to those obtained in phase 1 (the full list of p-values is provided in Supplementary Table S2).

**Table 2:** List of the mean values of the considered cardiovascular parameters in the subsequent phases of the pilot study.

Parameter	Phase						
	1	2	3	4	5	6	7
HR (bpm)	72.65	84.90^**^	67.69^†^	84.81^§^	67.65^§^	100.49^**^	64.12^‡^
SD	±12.01	±16.52	±8.05	±11.99	±11.02	±14.75	±10.00
Rel HR (%)	0	16.87^**^	−5.98^†^	17.68^§^	−6.70^§^	39.49^**^	−11.19^‡^
SD	0	±11.04	±7.18	±10.78	±4.51	±14.65	±8.65
IBI (ms)	856.07	742.87^**^	906.83^†^	725.21^§^	917.71^§^	614.53^**^	967.23^‡^
SD	±149.01	±138.30	±117.25	±97.43	±151.74	±88.87	±156.26
Rel IBI (%)	0	−13.15^**^	6.81^†^	−14.53^§^	7.42^§^	−27.58^**^	13.66^‡^
SD	0	±7.80	±7.59	±8.26	±5.43	±7.70	±11.44
SBP (mmHg)	123.39	117.62	126.08	130.47^†^	126.42	132.54^*^	131.63^*^
SD	±16.01	±14.52	±18.83	±20.68	±17.41	±25.57	±25.47
Rel SBP (%)	0	−4.30	2.85	5.96^†^	2.76	7.31^*^	6.37^*^
SD	0	±7.79	±14.69	±11.18	±10.42	±12.01	±10.58
DBP (mmHg)	76.31	73.31	71.40	90.02^†^	72.54	94.06^‡^	71.83
SD	±17.32	±11.11	±10.95	±11.71	±17.94	±11.83	±12.18
Rel DBP (%)	0	−1.11	−1.66	22.56^†^	−1.986	28.78^‡^	−1.99
SD	0	±17.76	±30.22	±28.18	±28.76	±33.02	±27.11
MAP (mmHg)	93.02	87.00	90.68	103.47^‡^	91.68	105.12^*^	91.93
SD	±17.14	±13.48	±13.41	±14.60	±15.44	±13.97	±15.17
Rel MAP (%)	0	−5.39	−0.70	12.73^‡^	−0.35	15.04^*^	0.21
SD	0	±11.64	±17.47	±14.19	±15.29	±18.52	±16.27
SV (ml)	88.98	69.36^*^	102.04	54.79^†^	105.38	42.65^§^	107.49^*^
SD	±32.77	±26.04	±24.60	±22.90	±65.52	±15.61	±38.31
Rel SV (%)	0	−19.75^*^	26.37	−34.09^†^	20.23	−47.50^§^	28.51^*^
SD	0	±26.43	±45.90	±30.00	±56.43	±25.05	±46.22
CO (l/min)	6.50	5.85^*^	7.03	4.58^*^	6.90	4.31^†^	7.03
SD	±2.34	±1.93	±2.42	±1.70	±3.95	±1.55	±2.66
Rel CO (%)	0	−7.16^*^	15.36	−25.49^*^	8.44	−29.52^†^	12.15
SD	0	±26.36	±40.13	±30.00	±52.04	±28.27	±36.89
LVET (ms)	325.25	255.28^**^	328.76	256.89^**^	331.55	241.8^§^	333.84
SD	±15.84	±26.47	±14.21	±22.77	±17.66	±34.65	±17.97
Rel LVET (%)	0	−21.45^**^	1.18	−20.87^**^	2.01	−25.35^§^	2.79
SD	0	±7.85	±3.96	±7.70	±4.51	±12.79	±5.99
RPP (mmHg/min)	0.92 × 10^4^	1.03 × 10^4†^	0.86 × 10^4^	1.11 × 10^4§^	0.86 × 10^4†^	1.36 × 10^4**^	0.87 × 10^4^
SD	±0.23 × 10^4^	±0.22 × 10^4^	±0.19 × 10^4^	±0.20 × 10^4^	±0.21 × 10^4^	±0.31 × 10^4^	±0.22 × 10^4^
Rel RPP (%)	0	12.28 × 10^4†^	−5.23 × 10^4^	23.07 × 10^4§^	−6.70 × 10^4†^	51.05 × 10^4**^	−3.43 × 10^4^
SD	0	±12.44 × 10^4^	±14.66 × 10^4^	±19.73 × 10^4^	±10.59 × 10^4^	±36.55 × 10^4^	±24.02 × 10^4^
TPR (MU)	1.20	1.03	0.91	1.62^*^	1.11	1.68	0.90^*^
SD	±0.61	±0.48	±0.33	±0.73	±0.46	±0.61	±0.35
Rel TPR (%)	0	−0.31	2.25	67.55^*^	6.14	74.28	−8.46^*^
SD	0	±49.98	±84.36	±103.12	±53.95	±101.34	±56.28

The significance levels for changes in the mean values obtained in the subsequent phases are shown in relation to those obtained in phase 1.

Rel - relative values calculated with reference to the initial level in phase 1.

MU - medical units.

Levels of significance.

^*^p ≤ 0.05; ^†^p ≤ 0.01; ^‡^p ≤ 0.005; ^§^p ≤ 0.0005; ^**^p ≤ 0.00005.

HR is one of the most essential parameters describing the hemodynamic state of the cardiovascular system and thus a commonly used indicator in clinical tests. Since the primary centre of the conduction system in the myocardium is under the influence of the autonomic nervous system (ANS), any changes to the inner, e.g. emotional state, and outer, e.g. changes in gravity, environment will be reflected in the ANS activity and therefore in the HR and IBI fluctuations.

During the rest (phase 1) mean HR in 17 subjects was HR = 72.6 bpm (beats per minute), SD = ±12.0, SE = ±2.9 (IBI = 856.1 ms, SD = ±149.0, SE = ±36.1), which is a normal value in a young person at rest. As a result of LBNP (phase 2), the arterial baroreceptors are unloaded and HR involuntarily increases in proportion to the intensity of stimulus and the amount of surface subject to it. HR increases to 84.9 bpm (increase by 16%), SD = ±16.5, SE = ±4.0 (IBI = 742.9 ms, i.e. decrease by 13%, SD = ±138.3, SE = ±33.5), which is accompanied by a slight increase in variance. This can be interpreted as the fact of small diversity in involuntary cardiac response in individual subjects. A stimulus of more than 1 minute duration triggers an additional vascular counterbalance mechanism that stabilises HR and increases TPR. This is why the termination of a stimulus is not accompanied by a decrease in HR to the initial values but slightly lower ones. This phenomenon is particularly visible in the panels showing relative changes (phases 3, 5, and 7). While there is no significant difference (p = 0.47) between a cardiac response to the LBNP stimulus (phase 2) and HUT (phase 4), an application of two stimuli at the same time (phase 6) results in a twofold increase in response to the stimulus and an enhanced reaction of return to balance (p ≤ 00005). No differences in mean HR were found between phases 3 and 5 (p = 0.81), whereas phase 7 demonstrated differences between mean HR at a level of p ≤ 0.05.

MAP is a controlled parameter in the cardiovascular system and is expressed as a product of CO and TPR. CO is in turn a product of HR and SV, hence MAP is given by

1 $${\rm{MAP}}={\rm{CO}}\times {\rm{TPR}}={\rm{HR}}\times {\rm{SV}}\times {\rm{TPR}}.$$

The resting value (phase 1) of SBP, DBP and MAP for 17 subjects was as follows: SBP = 123.4 mmHg, SD = ±16.0, SE = ±3.9, DBP = 76.3 mmHg, SD = ±17.3, SE = ±4.2, MAP = 93.0 mmHg, SD = ±17.1, SE = ±4.2. These are normal values in a young person at rest. The LBNP (phase 2), HUT (phase 4) tests and the complex stimulus (phase 6) cause blood pooling in the lower body, which leads to a decrease in venous return, and finally a decrease in SBP, DBP and MAP. The decrease in mean blood pressure in phase 2 is statistically of no significance (p = 0.09), unlike the increase in phases 4 and 6 (p ≤ 0.005 and p ≤ 0.05, respectively). Minor deviations in arterial blood pressure may be interpreted as an effect of the barostat, which is in fact a reflex loop from the arterial baroreceptors. The blood pressure regulation system efficiently eliminates the influence of a disturbing factor by maintaining the pre-set value, which does not mean that the blood pressure signal variation is not modified.

As mentioned above, the applied stimuli have direct influence over the amount of venous return. In the absence of reflective counterbalance this leads to a decrease in SV and CO. A modification of CO may be masked by a reflective change in HR. Since CO is a product of HR and SV, and changes in HR and SV are physiologically opposing, CO can remain at an unchanged level in spite of a stimulus disturbing the hemodynamic balance. The presented results regarding mean HR indicate a significant increase in HR proportional to the intensity of the stimulus used. The CO trend implies therefore the size of SV modification. Since the changes in SV are primal in relation to CO, they will be described first. The stimuli used cause a decrease in SV, with a trend of bigger changes being a function of the stimuli number. LBNP decreases SV by approximately 20%, HUT by around 40%, and LBNP with HUT causes a 50–60% decrease in SV in relation to its resting value.

The data analysis showed statistically significant differences in the mean values in individual test phases. A decrease in SV, in phase 2 (LBNP) was significant at a level of p ≤ 0.05. The comparison of mean values in phase 7 with phase 1 revealed significant differences at a level of p ≤ 0.05. But there is no difference in the mean values achieved in phases 3, 5 and 7, when we compare them with each other (p = 0.47). This indicates a slightly higher SV during rest after the stimulus (by around 20%), which is certainly related to a slower HR and a longer time needed to fill the ventricles. The changes in CO follow SV variations, except for phase 2, where the effect of LBNP was counterbalanced by an increase in HR, certainly due to a stepwise pressure reduction in the chamber.

SV is, in addition to HR, one of the most significant hemodynamic indicators detecting orthostatic intolerance. It can be argued that a decrease in SV by more than 60% can indicate the potential for an orthostatic collapse. Since this borderline value was not reached in any of the subjects, the above hypothesis needs verification. As CO is a combination of SV and HR, the monitoring of changes in CO can be essential to the evaluation of orthostatic (in)tolerance.

LVET is one of clinically used indicators of myocardial contractility and indirectly reflects the level of sympathetic activity. The ejection time is determined as the period from opening of the aortic valve to the closure of the aortic valve. The mean LVET values in all resting phases (1, 3, 5 and 7) did not differ in a statistically relevant way and remained at a level of 325.2 ms, SD = ±15.8, SE = ±3.8 (norm for a healthy person). Likewise, no statistically relevant differences were found in the mean values of ejection time for phases 2 and 4 (255.3 ms, SD = ±26.5, SE = ±6.4). The mean LVET during the complex stimulus (phase 6) was however significantly shorter in comparison with resting phases 1, 3, 5 and 7 (p ≤ 0.0005) as well as with phases of active single stimuli 2 and 4 (p ≤ 0.01 and p ≤ 0.0005, respectively), and it was 241.8 ms, SD = ±33.6, SE = ±8.4. Relative LVET changes in relation to the control condition achieved 20–25%. The behaviour of LVET under LBNP and/or HUT is already known, therefore in this paper we do not explain the mechanisms reflected by this parameter^19,20^.

Another indicator often used clinically is the so called double product defined also as the myocardial RPP coefficient. It is determined as a product of SBP and HR, i.e.

2 $${\rm{RPP}}={\rm{SBP}}\times {\rm{HR}}.$$

The mean resting phase (phase 1) RPP value was 9246 mmHg/min, SD = ±2263, SE = ±549, whereas it was slightly lower in the remaining resting phases (3, 5 and 7), i.e. approximately 8600 mmHg/min, where the difference was statistically not significant (p = 0.22). The RPP was increasing along with subsequent stimuli (phases 2 and 4) to reach a maximum value for the complex stimulus (phase 6). It is particularly evident in relative changes. The RPP in phase 2 was significantly (p ≤ 0.01) higher than in phase 1, i.e. 10,259 mmHg/min, SD = ±2176 SE = ±528. In phase 4, the mean RPP = 11,110 mmHg/min, SD = ±2040 SE = ±495 and was higher than resting RPP in a statistically relevant way (p ≤ 0.0005), and significantly higher than RPP in phase 2 (p ≤ 0.01). RPP in phase 6 reached a maximum (RPP = 13,596 mmHg/min, SD = ±3088 SE = ±749), which was a statistically relevant increase in relation to the resting value by 50% (p ≤ 0.00005). The trend of changes in the RPP observed during the test correlates with the intensity of the acting stimulus.

## Discussion

From a number of biomedical signals recorded, so far only the electrocardiogram and continuous blood pressure wave were selected to the analysis. The choice of signals and parameters calculated using those signals, very well known to physiologists concerned with tilt trials and LBNP tests, was deliberate. The latest literature reports show that the analysis of basic physiological parameters, e.g. heart rhythm and blood pressure, enables for predicting orthostatic hypotension^21^ and reveals precursory signs of syncope^22^. Our intention was to verify, in relation to specialist literature, the behaviour of a group of cadets experiencing accelerations on a regular basis who were subjected to tests in a device of a novel design. The pilot study confirmed the utility of the prototype of the ORTHO-LBNP system in tests of variable gravitational stimuli and highlighted its unique features such as high dynamics in changing tilt and negative pressure.

No symptoms of orthostatic collapse were observed in any subjects, which can be attributed to two causes. Firstly, the subjects are active pilots, which means that they have undergone a training improving their G-tolerance. Secondly, the stimuli could have not been adequate, i.e. too little loading, too short-lasting. A combination of tilt and LBNP stimulus can be considered that will destabilise the regulatory mechanisms of the cardiovascular system in future works.

The classification of efficiency of regulatory mechanisms in pilots, i.e. persons considered healthy, is still an extremely challenging task in aviation medicine. The tilt trial is very well known to physiologists in tests of the ANS, but require the patient to be observed during HUT for up to several dozen minutes or is finished at the moment when symptoms of orthostatic intolerance occur^23^. Classification tests should be kept as short as possible. This is why there are attempts to introduce indicators enabling a classification of patients according to physiological parameters measured on-line, during test. A known indicator of this type is the Crampton index (ICR)^24^, which takes into account the changes in HR and SBP, according to the equation:

3 $${\rm{ICR}}=25\times (3.15+0.1\times {\rm{\Delta }}\mathrm{SBP}-0.05\times {\rm{\Delta }}\mathrm{HR}),$$

where ΔSBP is the change in SBP, and ΔHR is the change in HR, produced by the HUT test. The classification is made according to the following criteria: 100 or more is the ideal value (rarely obtained), 95 and higher is regarded as very good, 80–94 as good, 65–79 as mediocre, 65 and less indicate poor condition or other health problems.

The Crampton index provides a general view on the “condition” of the autonomic circulatory regulation. Its simplicity and easiness to obtain are a considerable advantage, nonetheless they are also the main disadvantage. For there are a number of subtle factors making up the final picture of circulatory response which, according to the authors, should be among possible essential elements of the system’s description. Some researchers contested the use of heart rhythm and blood pressure as the only parameters for determining orthostatic tolerance also in the LBNP test area^25^. This is why, while addressing the needs, we suggest introducing an orthostatic tolerance index (OTI) which will, similarly to the Crampton index, quantify an examined person’s propensity to endure gravitational stimuli. We think that the OTI should take into account mean values of chosen physiological parameters and it can be generally written as

4 $${\rm{OTI}}={{\rm{C}}}_{1}+{{\rm{C}}}_{2}(\mathrm{HR},\mathrm{SV})+{{\rm{C}}}_{3}({\rm{SBP}},{\rm{DBP}},{\rm{TPR}})+{{\rm{C}}}_{4}({\rm{LVET}},{\rm{RPP}}),$$

where C_1_, C_2_, C_3_, and C_4_ are proportionality coefficients to be determined using tests carried out on a considerably larger group of examined pilots. An adequately large group of “average” persons, or just persons affected by vasovagal syndrome or generally by orthostatic intolerance, which will make it possible to determine statistically reliable proportionality coefficients and acceptable ranges of the changes including the limits of orthostatic intolerance.

Over many years of applying tilt tests^26,27^ and the LBNP method^28^, many devices of varied construction have been used. Most often tilt tables and LBNP chambers were separate devices used in independent experiments. As long as simple clinical trials were performed, manually operated tilt tables were sufficient. However, in recent years, precisely controlled drive systems, including robotic-assisted mechanisms, have been increasingly used in tilt tables for better control of the stimulus and to facilitate therapeutic treatments^29,30^. Underpressure generation systems have also been becoming more accurate, but the development of LBNP chambers has been somewhat different. Unlike tilt tables, LBNP chambers have been devices of high complexity since the beginning of their use, initially reserved for space medicine applications only^28,31^. The underpressure chambers designed for training in space exhibited an advanced level of construction that incorporated the latest achievements in material technology. With the increasing availability of composites and improving material processing, LBNP chambers have been launched to clinical practice^32–34^, rehabilitation and wellness^35^.

Only in a few solutions, a tilt table and a chamber for LBNP tests were integrated with each other. A simple device consisting of a manually adjustable tilt table and a chamber in which pressure was powered with a high power vacuum cleaner was presented by Protheroe *et al*.^36^. Their paper shows how to achieve a tilt in the range from −15° to +60° and a pressure of down to −60 mmHg at low cost. Those authors cite unpublished data from another research team, according to which predicted times to presyncope were determined in 63 subjects depending on gender and age group. They declared that unlike automatic tables, their solution allowed for rapidly transitioning from standing to supine position, however they did not list the maximum or typical rates of tilt changes achieved. Moreover, those authors attempt to argue that tilt testing with combined LBNP can be considered as a “gold standard” to study orthostatic intolerance. Although the tilt table testing is the most commonly applied test^37^, it has its limitations^38^. The main of them is the fact that it may be false positive, i.e. evoking complaints in subjects with a cardiac cause of syncope. Tilt testing (with or without LBNP) can therefore not serve as a clinical “gold standard” for estimating orthostatic tolerance. This diagnosis should incorporate clinical features as well.

Another device of simple construction was shown by Tymko *et al*.^39^. Those authors based their apparatus on a manually operated tilt table combined with a wooden hermetic box that acted as the LBNP chamber. An industrial vacuum with variable transformer was used to generate negative pressure. Although the table had the unique ability to place participants in varying body positions, from −90° to +90°, the experimental protocol included 45° HUT and HDT tests. 13 subjects undergone tilt tests and also LBNP trials at −50 mmHg. 3 participants achieved presyncope with application of LBNP in supine position, and 8 participants experienced presyncope in the 45° HUT position. As in the previous case, no values of the maximum rates of tilt and underpressure changes are known.

There are known works on restoring leg bone microvascular flow to supine levels by means of a HDT table integrated with a chamber to apply pressure of −25 mmHg to the subject’s lower body^40^. The table enables for manual tilting from 0° to −15°. The same custom tilting LBNP apparatus has been used previously to study intraocular pressure and transcranial ultrasound pulse amplitude^41^.

A concept based on two rims, similarly to that presented in model 5, was used in the device researched by another group^42^. During the study, 16 potential astronauts were examined with automatically adjusted tilt in the range from −70° to +70°, and LBNP reached −60 mmHg at the same time. The 17-minute combined HUT and graded LBNP paradigm led to provoked syncope in all of the subjects.

The market also offers a device with a motorised tilt table and a manually or automatically controlled LBNP chamber produced by *Physiology Research Instruments*. While the tilt is specified in the range from 0° to +65°, the authors have not been able to obtain information on the level of negative pressure generated.

Photographs of several other devices can be found in the literature^23^ and on the Internet, but no further data is available. In order to easily compare the proposed prototype system with other existing tilt tables with combined LBNP chambers, their most important features and research outcomes are shown in Table 3.

**Table 3:** List of the most important features of the tilt tables with combined LBNP chambers shown in the literature and the main results of research works carried out with them.

Features			Subjects; experimental procedure; main outcome	Reference
Tilt table	LBNP chamber	Biomedical monitoring		
Manually adjustable in the range from −15° to + 60° with 10° increment, steel frame, adjustable right-armrest, adjustable footplate	Manually adjustable down to −60 mmHg, high power vacuum cleaner, plastic and wooden elements, neoprene waist seal	ECG, blood pressure, brachial and cerebral blood flow, end-tidal tensions of oxygen (P_ET_O_2_) and carbon dioxide (P_ET_CO_2_)	No information on number of subjects; 20-minute supine rest, 20-minute 60° HUT at 30-second transition to upright, 3 sequences of 10-minute HUTs and LBNP at −20, −40 and −60 mmHg, respectively, recovery; suggestion that tilt testing with combined LBNP can be considered as a “gold standard”	Protheroe *et al*.^36^
Manually adjustable in the range from −90° to + 90°, steel frame	Manually adjustable down to −50 mmHg, industrial 6-horsepower vacuum, wooden box, kayak spray skirt	ECG, HR, blood pressure, MAP, SV, CO, P_ET_CO_2_, transcranial Doppler, cerebral blood velocity (CBV)	13 subjects (males); 5-minute supine rest, 10-minute −50 mmHg supine LBNP, 15-minute recovery, 10-minute 45° HDT or −45° HUT and −50 mmHg LBNP, recovery; body position has no effect on CBV responses during LBNP, CBV decreases during LBNP	Tymko *et al*.^39^
Manually adjustable in the range from 0° to −15°, steel frame	Manually adjustable down to −25 mmHg, industrial vacuum pump, steel and Plexiglas elements, neoprene seal	Photoplethysmography, tibial and skin blood flows, NIRS	11 subjects (8 males and 3 females); 5-minute sitting on the tilt table, 5-minute supine rest, 5-minute −15° HDT, 10-minute −15° HDT and −25 mmHg LBNP; short-term LBNP can reduce the HDT-induced increase in microvascular blood flow in the tibia to values consistent with sitting posture	Siamwala *et al*.^40^
Automatically adjustable in the range from −70° to + 70°, table attached to the movable steel rim, adjustable footplate	Automatically adjustable down to −60 mmHg by an advanced subsystem for generating underpressure, steel and Plexiglas elements, neoprene seal	ECG, HR, blood pressure, MAP, SBP, DBP, impedance cardiography, SV	15 subjects (males); 30-minute supine rest, 5-minute 70° HUT followed by additional 20 mmHg LBNP that is increased by additional 10 mmHg LBNP every three minutes until presyncope occurred; mean standing time from begin HUT to presyncope was determined	Grasser *et al*.^42^
Automatically adjustable in the range from −45° to + 80° with up to 45°/s rate of tilt changes, steel frame, adjustable handrest, adjustable footboard	Automatically adjustable down to −100 mmHg with up to 20 mmHg/s rate of underpressure changes, Al and Plexiglas elements, neoprene waist seal	ECG, HR, IBI, blood pressure, MAP, SBP, DBP, SV, CO, LVET, RPP, TPR, pulse oximetry, EMG, SCL, body temperature, impedance reography, EEG, respiration curve, NIRS	17 subjects (14 males and 3 females); 5-minute supine rest, −100 mmHg supine LBNP, 75° HUT, −60 mmHg LBNP associated with 75° HUT, separated by 3-minute resting phases; recovery; the prototype system can map any pre-programed tilt and LBNP profiles, confirmation of the efficiency of performing experimental procedures	Dziuda *et al*.

With regard to the aforementioned devices, the prototype of the ORTHO-LBNP system is technologically advanced equipment that meets the high demands of the aviation simulators. The only competitor could be the two-rim-based device due to its innovative drive system^42^. However, given the technical parameters our prototype system offers higher performance in terms of the tilt range and the level of generated underpressure. It is difficult to relate to dynamics, as none of the devices have been characterised in this regard.

### Future work

The novel system provides the opportunity to carry out experimental procedures in a much wider range than described above. The authors are planning to extend the current analysis to a bigger cohort in the future and consider gender comparison to assure population wide acceptability of the developed system. Data from a larger population of pilots will also allow for differentiating between pilots of fighter aircraft, transport aircraft and helicopters. Efforts will also be made to work out the final form of the OTI.

The effect of muscle pump on facilitating venous return and degree of blood pooling achieved during orthostatic challenge was not measured. Astronauts returning from long duration spaceflight often experience orthostatic intolerance due to cardiovascular deconditioning and skeletal muscle atrophy^43,44^. Role of muscle pump is pivotal during orthostatic challenge to facilitate venous return and thus regulate blood pressure^45^. Therefore, in the future study the authors will also analyse skeletal muscle activity in response to orthostatic challenge evoked by the ORTHO-LBNP system to comprehensively validate its potential as a system to mitigate physiological deconditioning. Furthermore, our intention is to use NIRS to accurately measure blood volume in the calf musculature^46^.

## Methods

### Experimental protocol

The experimental protocol complied with the Declaration of Helsinki, and was approved by the Ethics Committee of the Military Institute of Aviation Medicine (Decision 18/2015), in which the study was carried out. The pilot study was registered at https://clinicaltrials.gov/ on 27/11/2017 under number NCT03354234. All activities during the study were performed in accordance with the relevant guidelines and regulations. The subjects were informed in detail on the purpose and nature of the study using the prototype of the ORTHO-LBNP system and signed their informed consent for study participation and publication of identifying information/images, including online open-access publications.

The pilot study was carried out in an isolated room in the presence of a physician (physiologist), a paramedic and an ORTHO-LBNP system operator. The paramedic prepared the subject for the study by placing on his/her body electrodes and measuring sensors that were removed after completion of the study. The subject was asked to close his/her eyes during the tests and to place his/her hands on the rest. In order to cause the least disturbance, no conversation was held with the subject. Only if disturbing symptoms occurred, was the subject to immediately report this to the personnel. The physician was systematically observing the subject’s cardiogram, his/her HR, CNAP wave, SBP and DBP in comparison with the changing tilt of the table and negative pressure in the chamber. Any time, at the request of the subject or on the instruction of the physician, the operator could interrupt the tests. The tilt table then returned to the Trendelenburg position if interruption had occurred during HUT and/or LBNP stimuli, or to the horizontal position if interruption had occurred during HDT stimulus. At the same time atmospheric pressure was resumed in the chamber.

### Physiological monitoring

**Figure 6:**
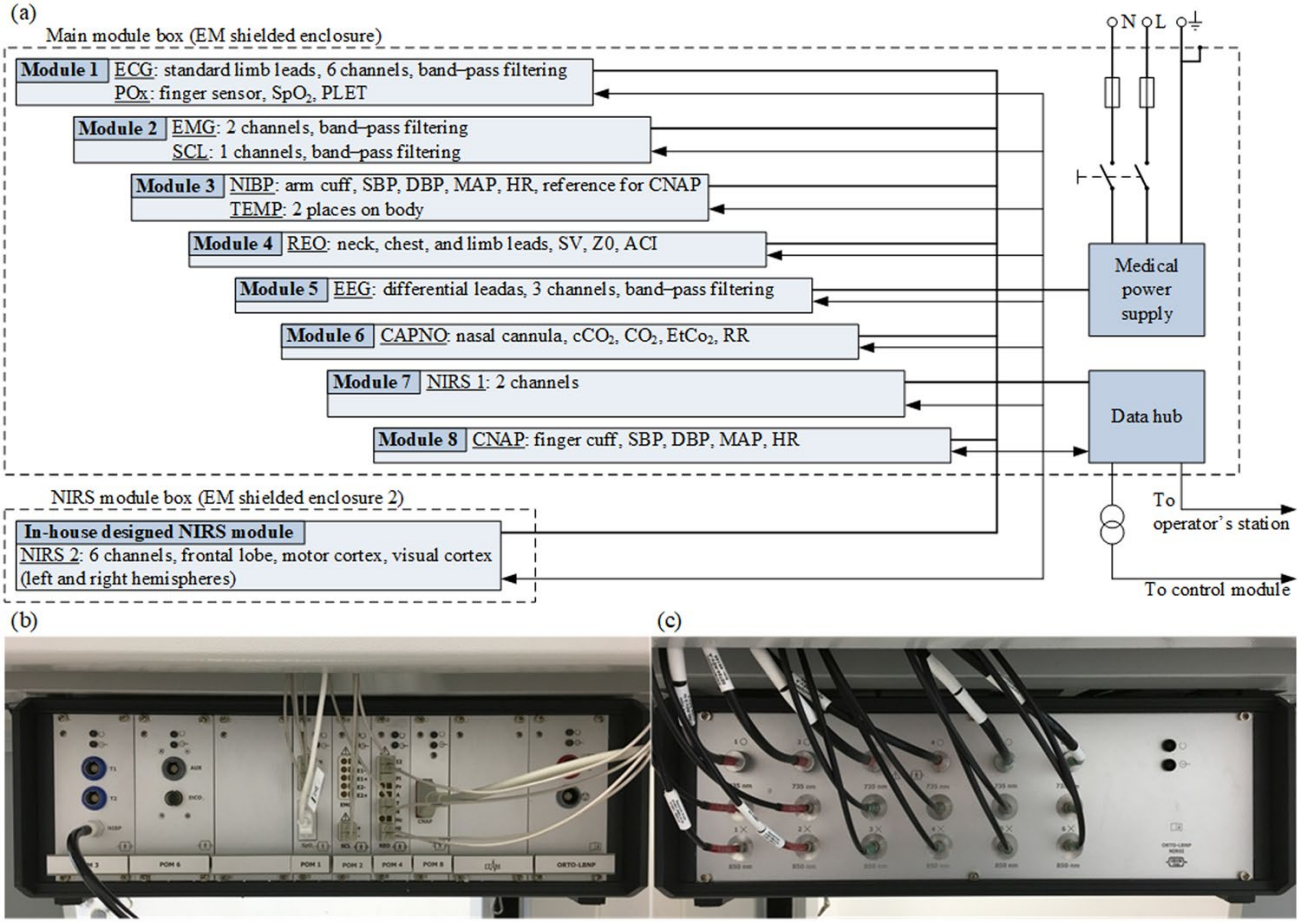
Medical monitoring subsystem: the (**a**) block diagram, (**b**) main module box by the *Institute of Medical Technology and Equipment*, Poland, (**c**) in-house-designed module box by the *Nałęcz Institute of Biocybernetics and Biomedical Engineering of the Polish Academy of Sciences*.

A subsystem for monitoring physiological parameters in subjects undergoing tilt and underpressure tests is necessary to carry out examinations safely and to classify subjects reliably. The authors decided to build their own measurement subsystem where all the medical recorders in the form of modules were integrated in electromagnetically (EM) shielded enclosures to which the wires from the electrodes and sensors located on the subject’s body were connected as in modern operating tables. Figure 6(a) shows the general configuration of the subsystem that consists of modules for the following types of diagnostics: ECG, pulse oximetry (POx), electromyography (EMG), skin conductance level (SCL), NIBP, body temperature (TEMP), impedance reography (REO), electroencefalography (EEG), capnography (CAPNO), near-infrared spectroscopy (NIRS) and CNAP. The modules for measuring ECG, EMG, SCL, TEMP, REO and EEG were developed entirely by the engineering team, whereas the POx (WW3711 by *Smith Medical*), NIBP (Advantage Mini by *SunTech Medical*), CAPNO (miniMediCO2 by *Oridion Medical*), CNAP (CNAP by *CNSystems Medizintechnik AG*) and NIRS (7610 OEM by *Nonin*) modules were built on the basis of commercially available certified OEM solutions. In addition to the commercial off-the-shelf 2-channel NIRS device, we used an in-house-designed 6-channel module to record oxygenation changes in the left and right frontal lobe, motor cortex, and visual cortex. All the modules were connected to the operator’s station and the control module via the data hub. The module set and data hub were closed in a propacPRO series EM shielded enclosure by *Schroff* (Fig. 6(b)), but the in-house-designed NIRS module was enclosed separately in another propacPRO housing due to its size (Fig. 6(c)). The medical monitoring subsystem has been certified with a CE marking indicating conformity with the standards harmonised with Directive 93/42/EEC concerning medical devices.

### Prototype system

Before constructing the prototype system, models of the LBNP chamber, tilt table, and monitoring subsystem were built. As far as the model of the medical monitoring subsystem became part of the prototype of the ORTHO-LBNP system, the laboratory evaluation and optimisation work led to the development of a separate version of the tilt table with LBNP chamber. The improvements mainly focused on the construction durability and a design of the table frame that would withstand both the weight of the medical monitoring subsystem fixed under the tilt table, the LBNP chamber and the patient laying inside the chamber. Both in the model and prototype system, the 1-kW HF-SP102B servo motor with an integrated table motion controller and angular planetary gearbox by *Mitsubishi Electric* were used to drive the tilt table. Similarly, the 0.85-kW SC401MG side channel blower by *Emmecom* was verified in the model and then used in the prototype system to generate underpressure. As the blower generates noise at the level of 63 dB at maximum rotations, it was built in to a soundproofed casing to reduce noise by approximately 30% and to ensure comfort to subjects.

The tilt table and LBNP chamber underwent tests for compatibility with the harmonised standards in regards to machinery Directive 2006/42/EC, and hence these two mechanical subsystems have been CE marked. Moreover, the safety of the prototype system has been confirmed by the positive results of laboratory tests carried out with respect to Directive 93/42/EEC.

A computer application for controlling the tilt table and LBNP chamber, as well as receiving, analysing and presenting data from the medical modules during examination was developed during construction of the prototype system. The software runs on the *Microsoft*. Net Framework 4.0 platform. It was prepared in the C # language using the Visual Studio environment. The source code was developed according to the applicable design principles and software engineering principles. A set of tests for the individual software units and integration tests were also prepared. Similar to the medical monitoring subsystem, the computer application complies with the requirements of the standards harmonised with Directive 93/42/EEC and has been certified with a CE marking.

**Figure 7:**
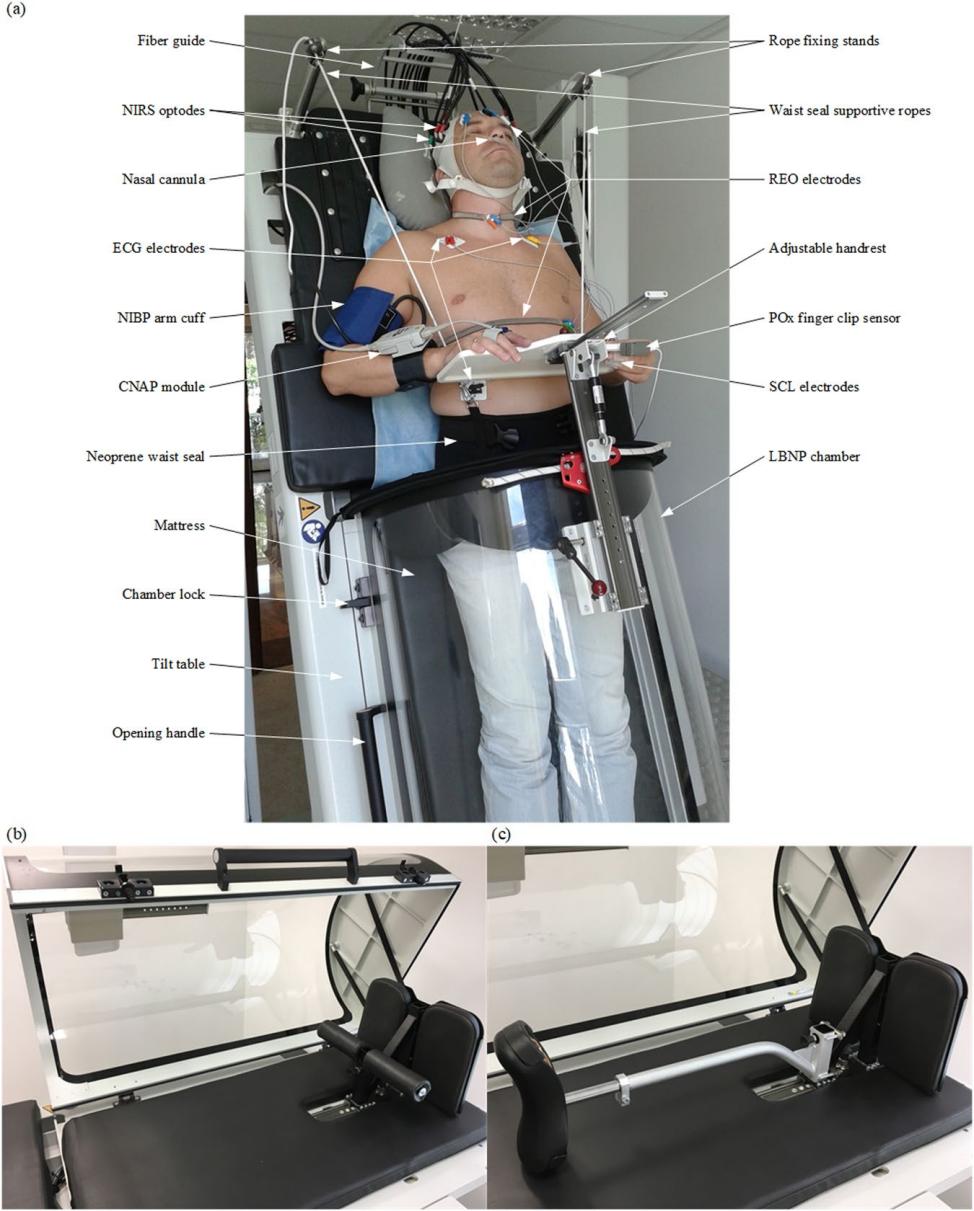
Photographs of the prototype system: a (**a**) subject during an examination, the (**b**) open LBNP chamber with the footboard, (**c**) optionally mounted saddle.

Finally, the prototype system developed at technology readiness level 9 was deployed in a room with medical approvals, and prepared for pilot-based studies. A photograph of a subject being examined with the prototype of the ORTHO-LBNP system is shown in Fig. 7(a). A neoprene skirt similar to those used in kayak sports was applied to seal the space between the subject and the edge of the LBNP chamber. The sealing skirt was customised by the manufacturer so that two ropes could be attached to it to prevent the skirt from sliding off the hips when negative pressure was applied. The ropes were fastened on the other side to the stands mounted at the head of the table. For a continuous recording of arterial pressure acquired from the subject’s finger at the heart level, an adjustable handrest was designed and installed on top of the LBNP chamber.

A unique feature of the prototype system is the ability to carry out experiments with a saddle on which the patient is placed for minimising the impact of the footboard (Fig. 7(b)) on the subject’s legs. In this way some researchers tried to reduce the leg muscle tension and weaken the blood pushing out toward the upper part of the body^47–49^. Thus the seat may intensify the effect of blood pooling in the lower extremities and we decided that the prototype system would be equipped with a removable saddle as an option for research applications, as shown in Fig. 7(c).

### Statistical analysis

For each of the physiological parameters extracted from the ECG and CNAP signals, descriptive statistics were determined in relation to the relevant experiment phase. Then, the normality of the distribution of the mean values was tested for all the subjects. A negative result of the normal distribution test with regard to most data, i.e. a mixed character of this distribution, triggered the need for using nonparametric statistical tests. The mean values were therefore compared through a nonparametric Friedman-Kendall’s analysis of variance for multiple dependent samples^50^ using *Statistica*. The significance level was set at p ≤ 0.05. The p-value reported in Table 2 is a comparison of different phases with the initial level in phase 1.

## Electronic supplementary material

Supplementary Information

Physiological Data

Statistical Data

Supplementary Video
