# Supplementary material for: Development and evaluation of a novel system for inducing orthostatic challenge by tilt tests and lower body negative pressure
Source: Sci Rep. 2018 May 17;8:7793. doi: 10.1038/s41598-018-26173-2 (PMC5958117; doi:10.1038/s41598-018-26173-2)
Supplement: Supplementary file 1 — Supplementary Information [file 41598_2018_26173_MOESM1_ESM.pdf]

Supplementary Information

Development and evaluation of a novel system for inducing orthostatic challenge by tilt tests and lower body negative pressure

Łukasz Dziuda[1,*], Mariusz Krej[1], Maciej Śmietanowski[2], Aleksander Sobotnicki[3], Mariusz Sobiech[3], Piotr Kwaśny[4], Anna Brzozowska[4], Paulina Baran[1], Krzysztof Kowalczuk[5], Franciszek W. Skibniewski[1]

[1] Department of Flight Simulator Innovations, Military Institute of Aviation Medicine, ul. Krasińskiego 54/56, 01-755 Warszawa, Poland

[2] Department of Experimental and Clinical Physiology, Medical University of Warsaw, ul. Banacha 1B, 02-097 Warszawa, Poland

[3] Department of Research and Development, Institute of Medical Technology and Equipment, ul. Roosevelta 118, 41-800 Zabrze, 41-800, Poland

[4] ETC-PZL Aerospace Industries Sp. z o.o., Aleja Krakowska 110/114, 02-256 Warszawa, Poland

[5] Department of Simulator Studies and Aeromedical Training, Military Institute of Aviation Medicine, ul. Krasińskiego 54/56, 01-755 Warszawa, Poland

[*] Corresponding Author (ldziuda@wiml.waw.pl)

Table of Contents

Figure S1. Model 1 of the tilt table: (a) general structure, (b) drive method. 4
Figure S2. Model 2 of the tilt table: (a) general structure, (b) drive method. 5
Figure S3. Model 3 of the tilt table: (a) general structure, (b) drive method. 6
Figure S4. Model 4 of the tilt table: (a) general structure, (b) drive method. 7
Figure S5. Model 5 of the tilt table: (a) general structure, (b) drive method. 8
Figure S6. Model 6 of the tilt table: (a) general structure, (b) drive method. 9
Figure S7. Trajectories of the body on the tilt table during (a) HUT and (b) HDT tests. 10
Figure S8. Concept of the pneumatic subsystem for generating underpressure. 11
Figure S9. Strength analysis of the chamber based on an Al frame, 1 transverse beam, 30° slope, PC inserts, 5 mm thick 12
Figure S10. Strength analysis of the chamber based on an Al frame, 2 transverse beams, 45° slope, PC inserts, 6 mm thick 13
Figure S11. Strength analysis of the chamber based on a thermoformed PC sheet, 8 mm thick 14
Figure S12. Strength analysis of the chamber based on a thermoformed corrugated PC sheet, 6 mm thick. 15
Figure S13. The prototype of the ORTHO-LBNP system: (a) *Autodesk Inventor* model and (b) its physical realisation. 16
Figure S14. Block diagram of the medical monitoring subsystem. 17
Figure S15. Medical monitoring subsystem: the (a) main module box by the *Institute of Medical Technology and Equipment*, Poland, (b) in-house-designed NIRS module box by the *Nałęcz Institute of Biocybernetics and Biomedical Engineering of the Polish Academy of Sciences*. 18
Figure S16. Block diagram of the prototype system. 19
Figure S17. Subject during an examination using the prototype system. 20
Figure S18. Photographs of the prototype system: (a) open LBNP chamber with the footboard, (b) optionally mounted saddle. 21
Figure S19. Example of an original recording: table tilt, LBNP profile, ECG signal, and CNAP wave. 22
Figure S20. Traces of the physiological parameters extracted from the ECG and CNAP signals in the subsequent phases of the pilot study. 23
Figure S21. (a) Absolute and (b) relative (in relation to the initial level in phase 1) changes in HR in the subsequent phases of the pilot study. 24
Figure S22. (a) Absolute and (b) relative changes in IBI in the subsequent phases of the pilot study. 25
Figure S23. (a) Absolute and (b) relative changes in SBP in the subsequent phases of the pilot study 26
Figure S24. (a) Absolute and (b) relative changes in DBP in the subsequent phases of the pilot study 27
Figure S25. (a) Absolute and (b) relative changes in MAP in the subsequent phases of the pilot study 28
Figure S26. (a) Absolute and (b) relative changes in SV in the subsequent phases of the pilot study. 29
Figure S27. (a) Absolute and (b) relative changes in CO in the subsequent phases of the pilot study. 30
Figure S28. (a) Absolute and (b) relative changes in LVET in the subsequent phases of the pilot study 31

Figure S29. (a) Absolute and (b) relative changes in RPP in the subsequent phases of the pilot study....32
Figure S30. (a) Absolute and (b) relative changes in TPR in the subsequent phases of the pilot study....33

Table S1. Results of the tilt table construction optimisation....34
Table S2. List of the mean values of the considered cardiovascular parameters in the subsequent phases of the pilot study. The significance levels for changes in the mean values obtained in the subsequent phases are shown in relation to those obtained in phase 1. 35
Table S3. List of the most important features of the tilt tables with combined LBNP chambers shown in the literature and the main results of research works carried out with them....36

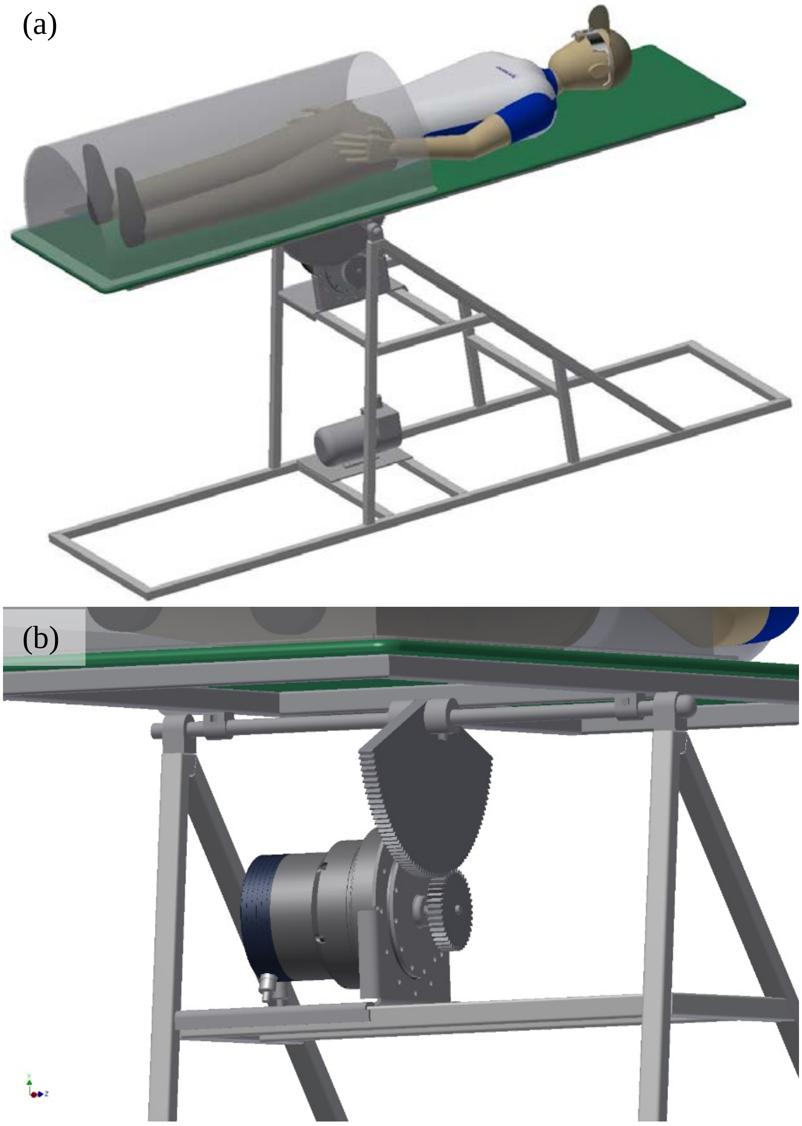

Figure S1. Model 1 of the tilt table: (a) general structure, (b) drive method.

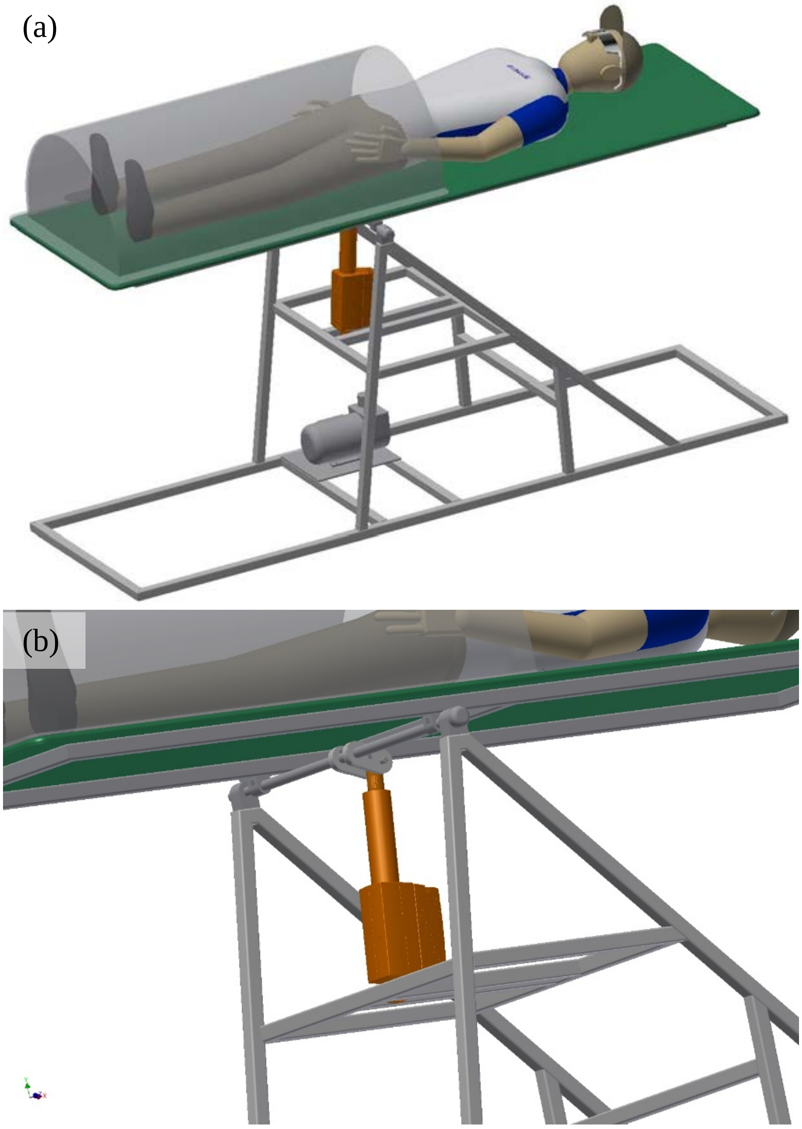

Figure S2. Model 2 of the tilt table: (a) general structure, (b) drive method.

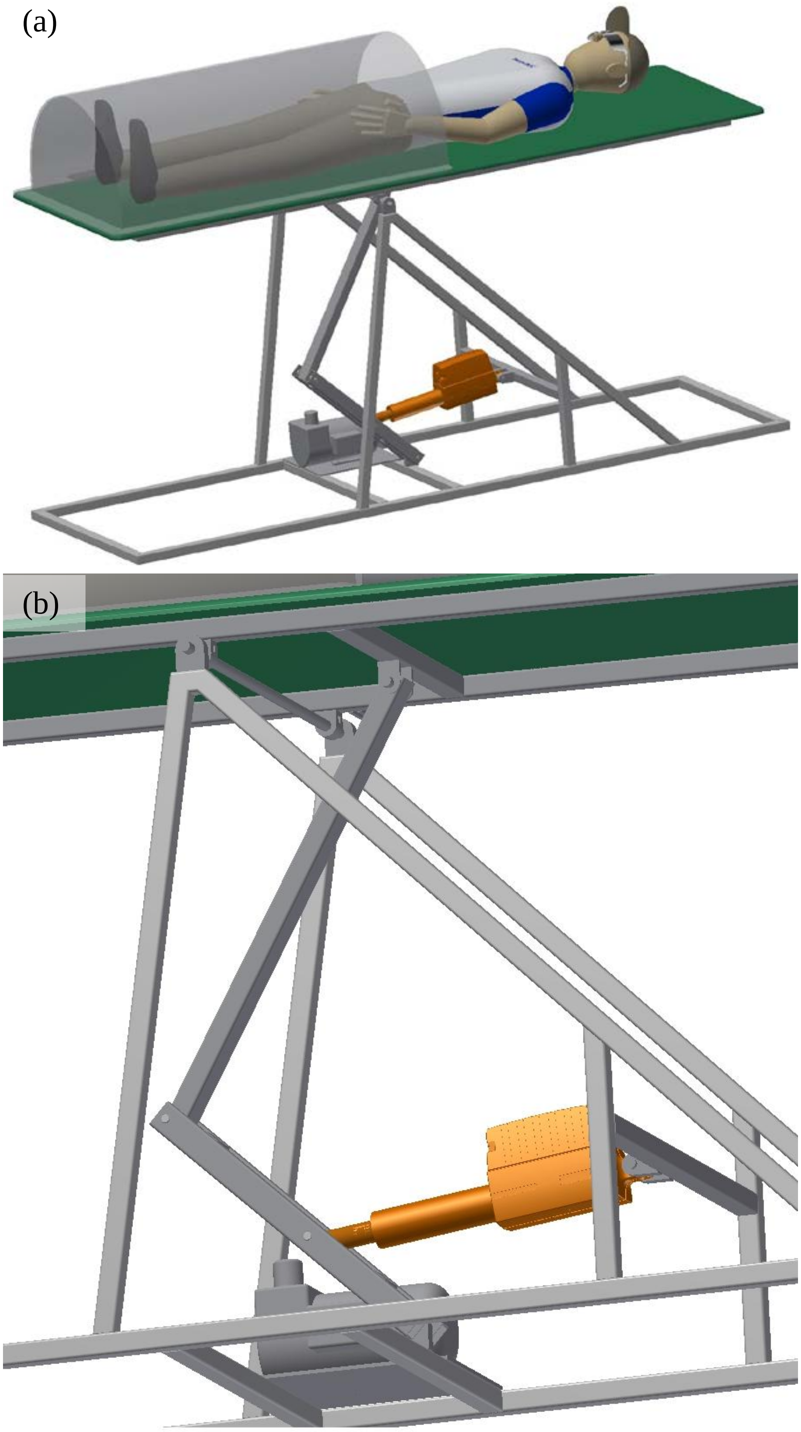

Figure S3. Model 3 of the tilt table: (a) general structure, (b) drive method.

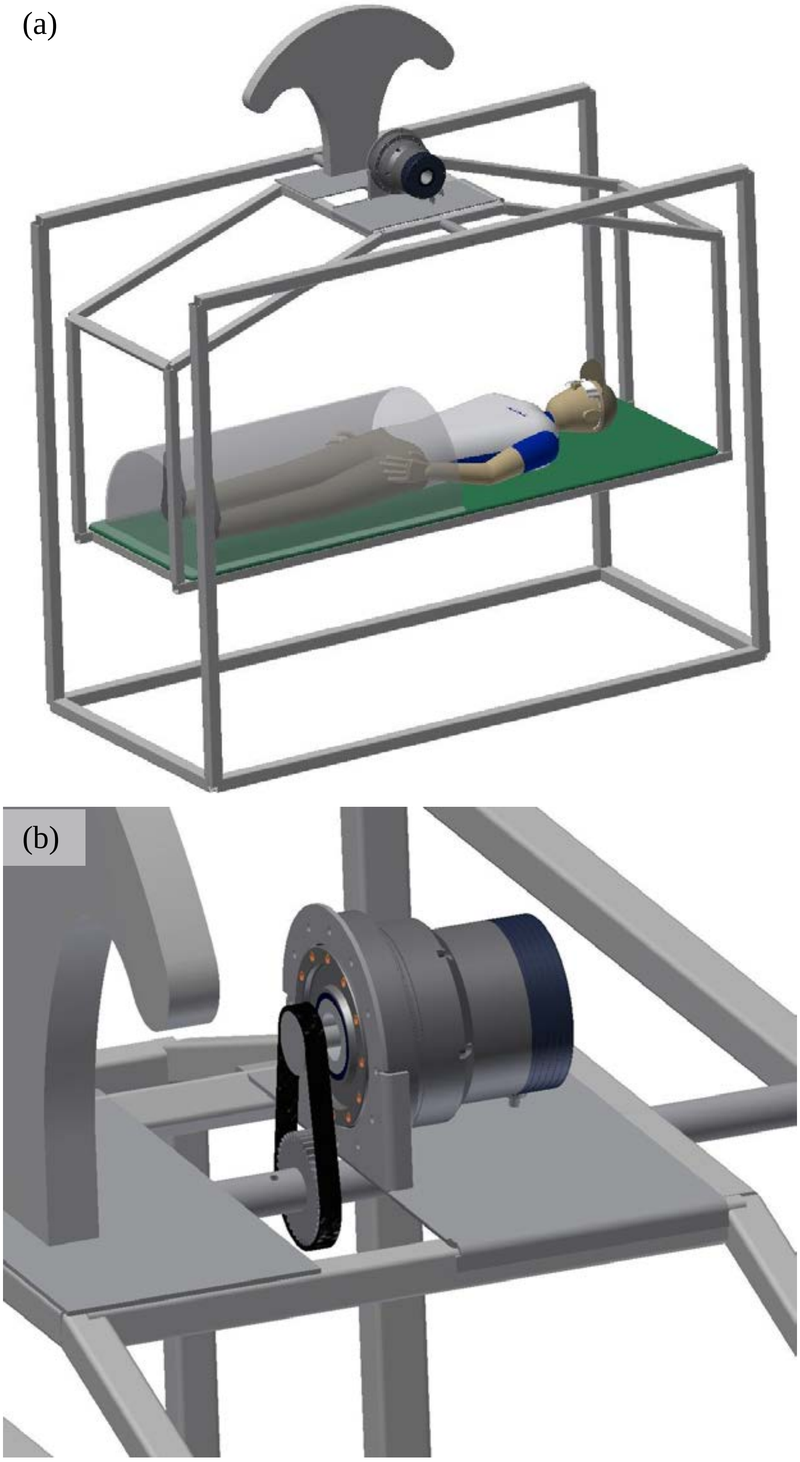

Figure S4. Model 4 of the tilt table: (a) general structure, (b) drive method.

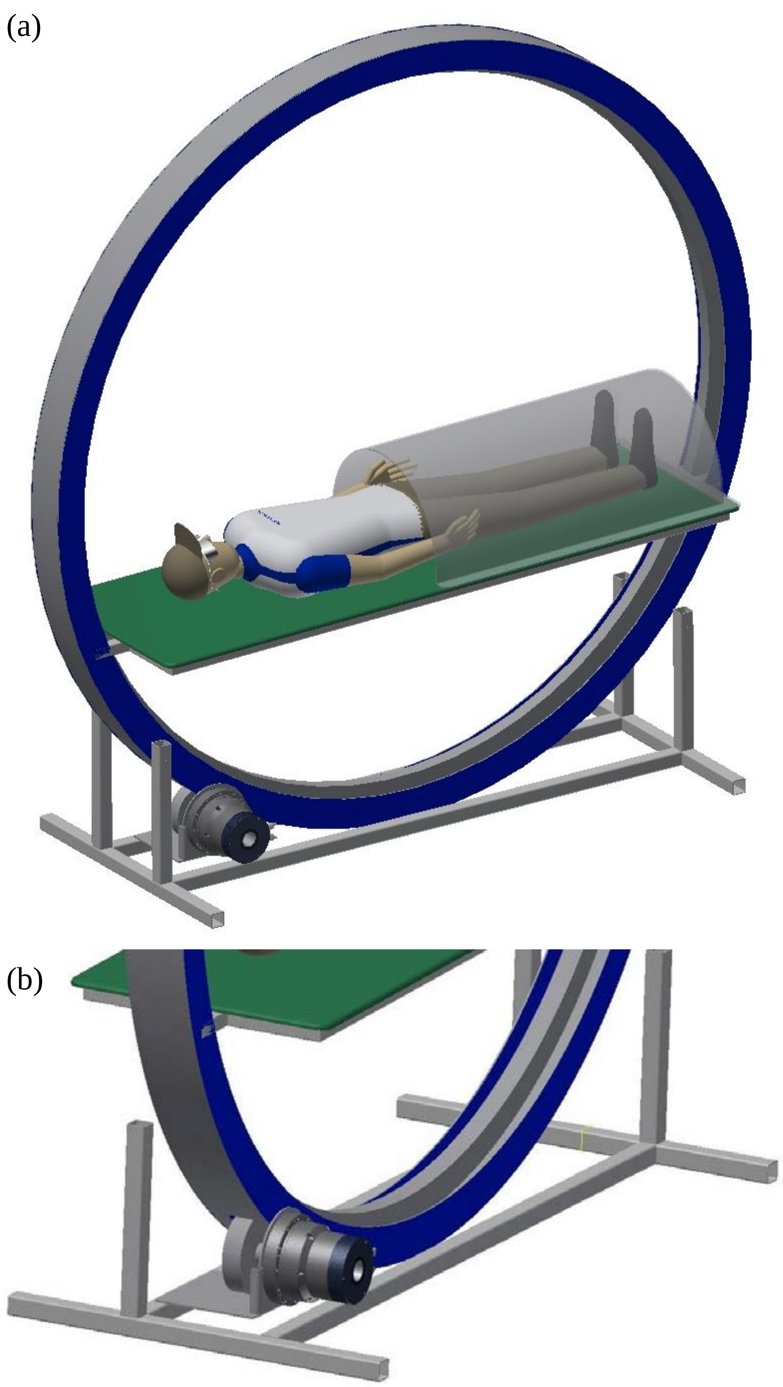

Figure S5. Model 5 of the tilt table: (a) general structure, (b) drive method.

(a)

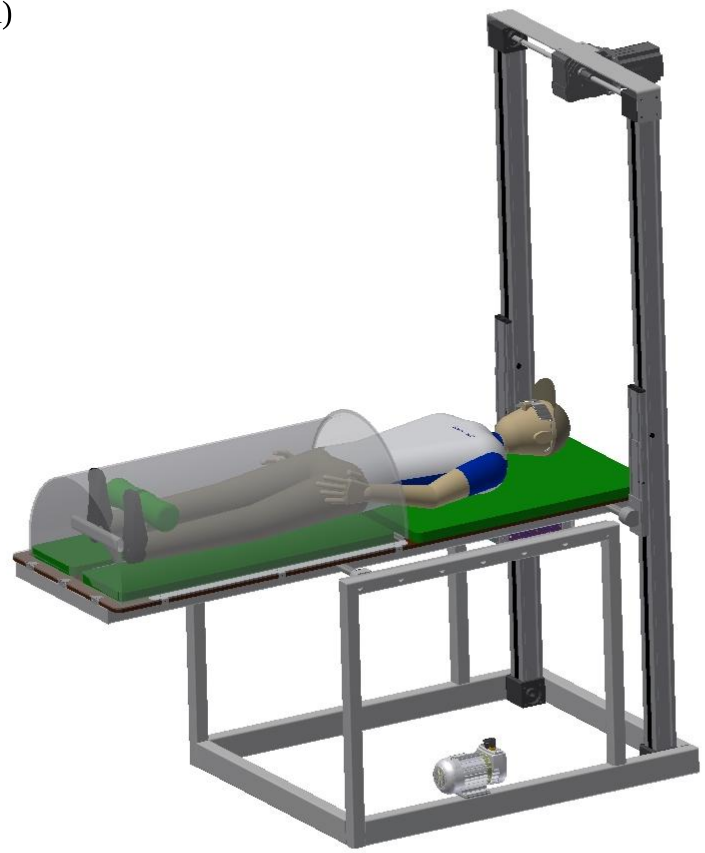

(b)

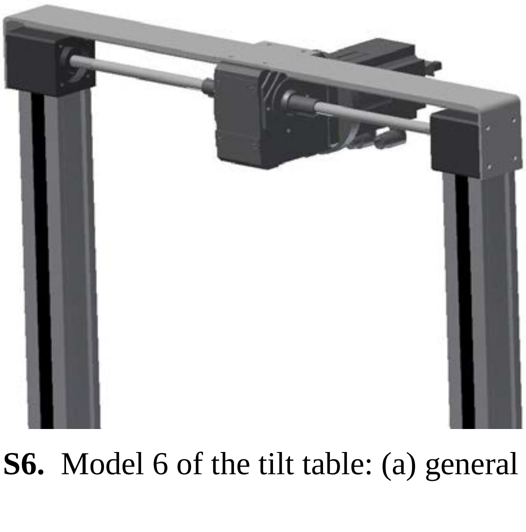

Figure S6. Model 6 of the tilt table: (a) general structure, (b) drive method.

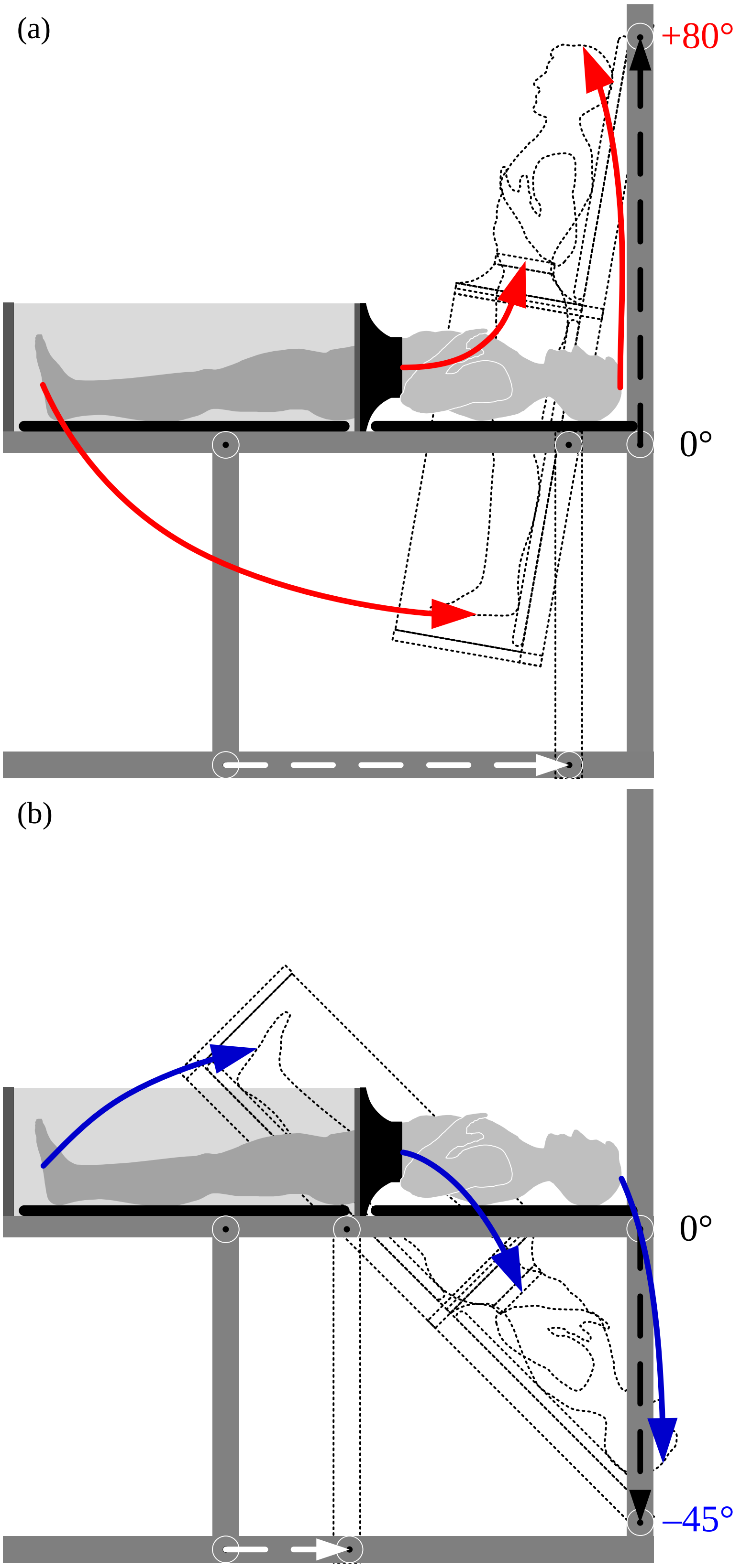

Figure S7. Trajectories of the body on the tilt table during (a) HUT and (b) HDT tests.

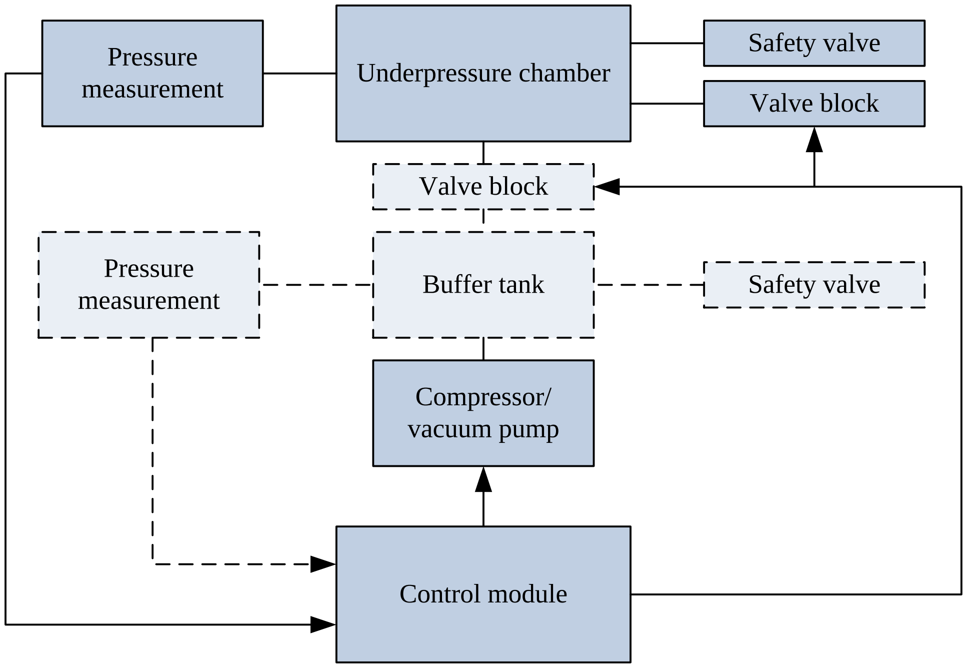

Figure S8. Concept of the pneumatic subsystem for generating underpressure.

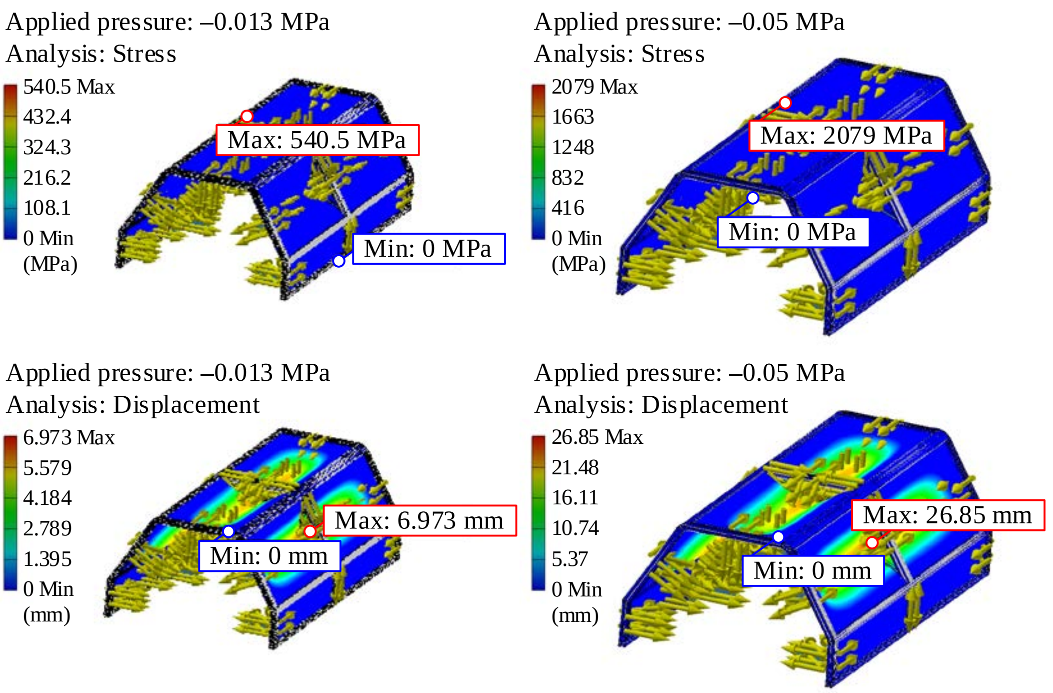

Figure S9. Strength analysis of the chamber based on an Al frame, 1 transverse beam, 30° slope, PC inserts, 5 mm thick.

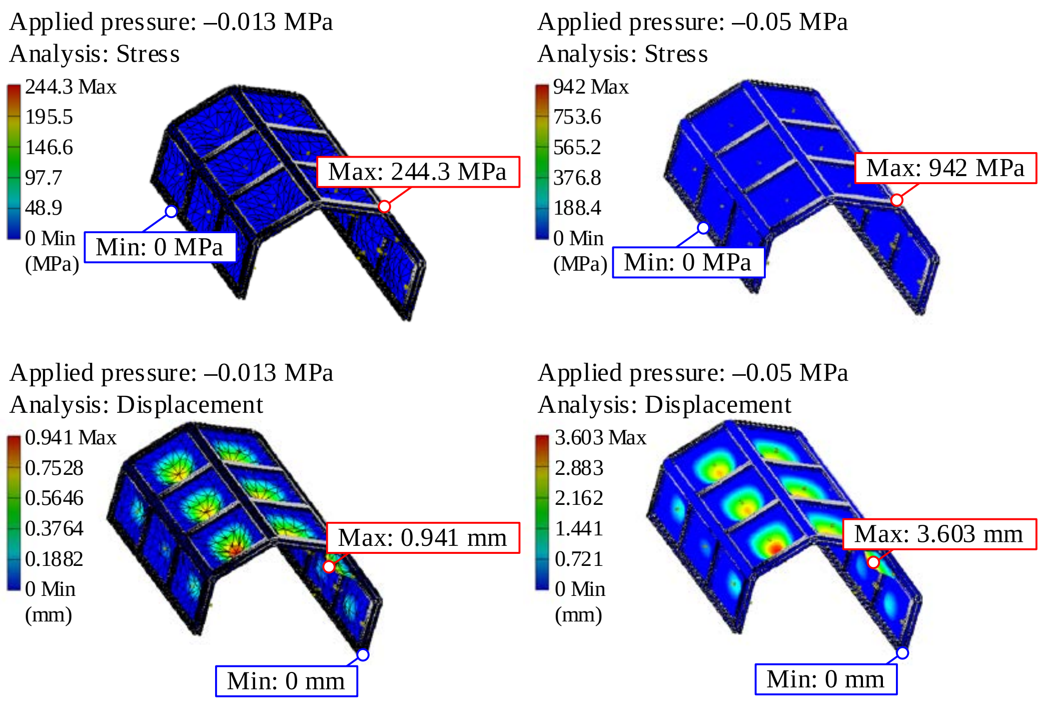

Figure S10. Strength analysis of the chamber based on an Al frame, 2 transverse beams, 45° slope, PC inserts, 6 mm thick.

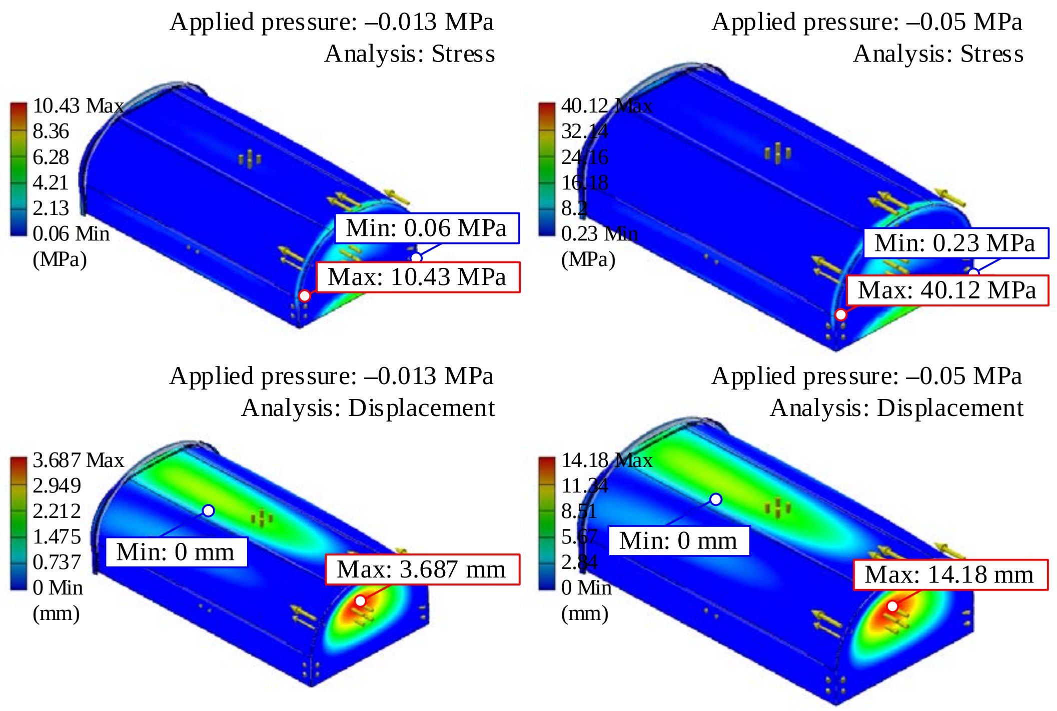

Figure S11. Strength analysis of the chamber based on a thermoformed PC sheet, 8 mm thick.

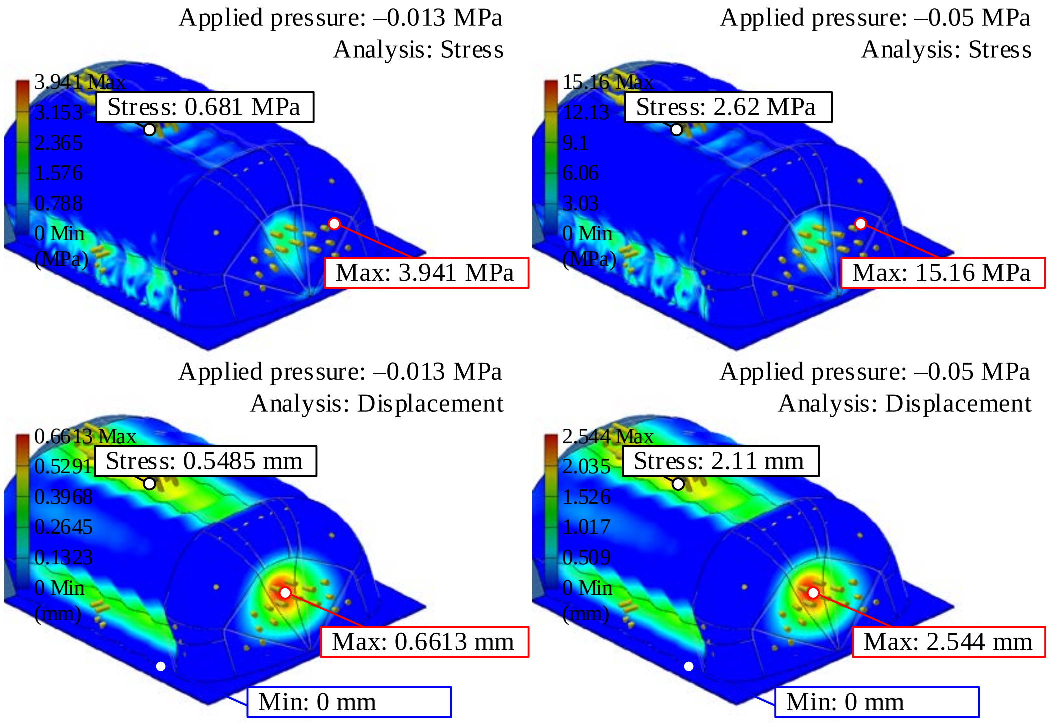

Figure S12. Strength analysis of the chamber based on a thermoformed corrugated PC sheet, 6 mm thick.

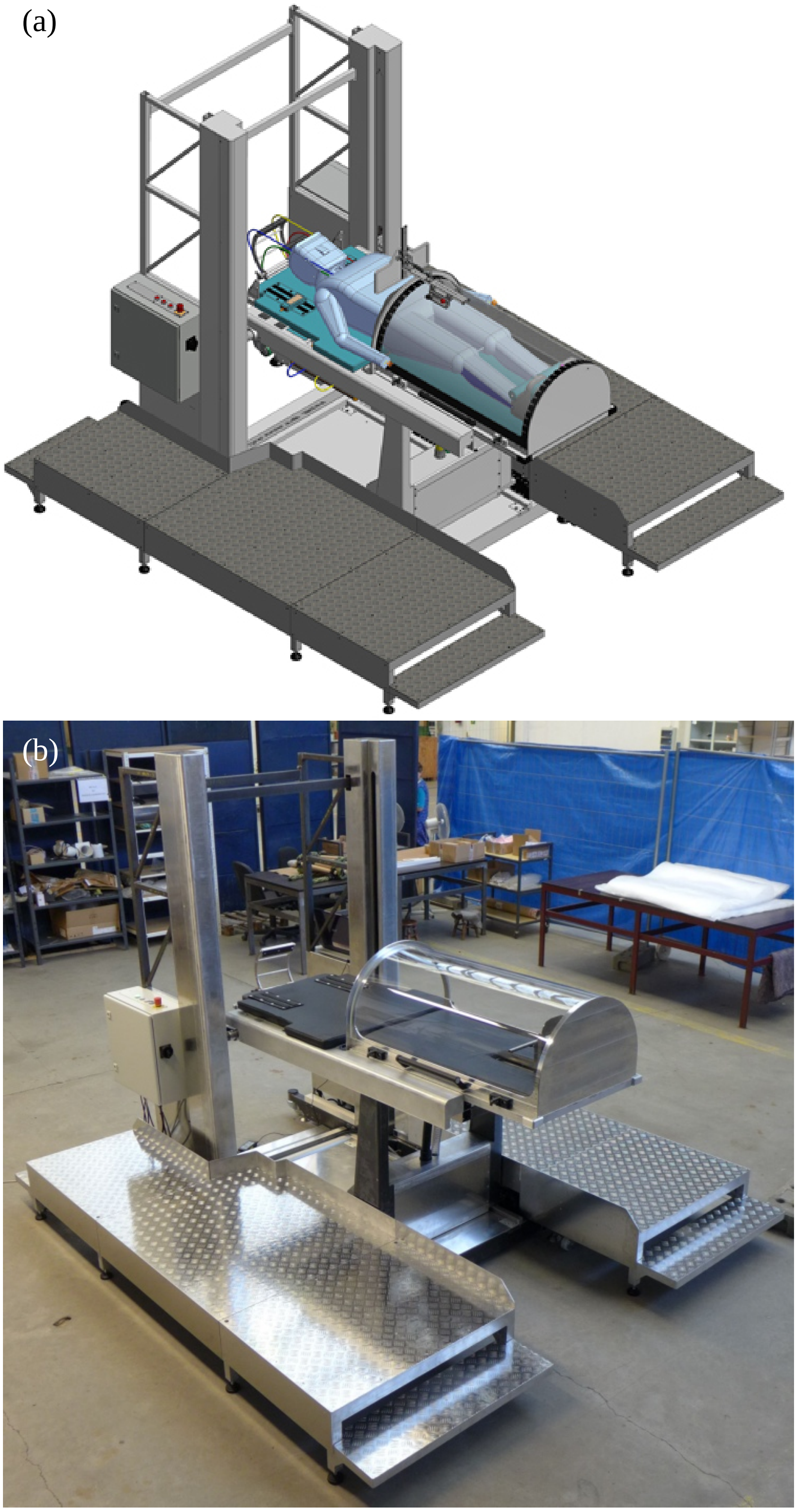

Figure S13. The prototype of the ORTHO-LBNP system: (a) *Autodesk Inventor* model and (b) its physical realisation.

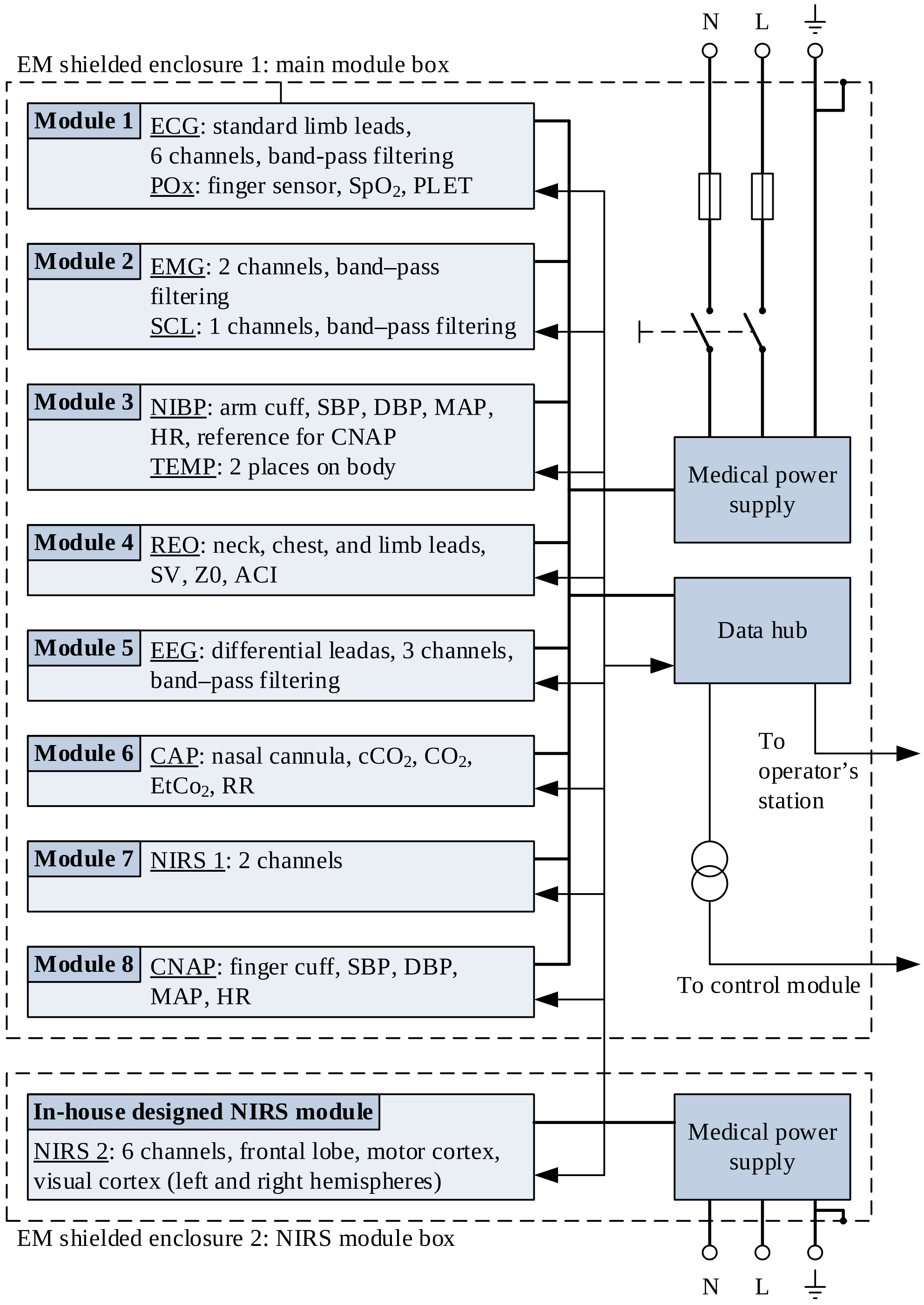

Figure S14. Block diagram of the medical monitoring subsystem.

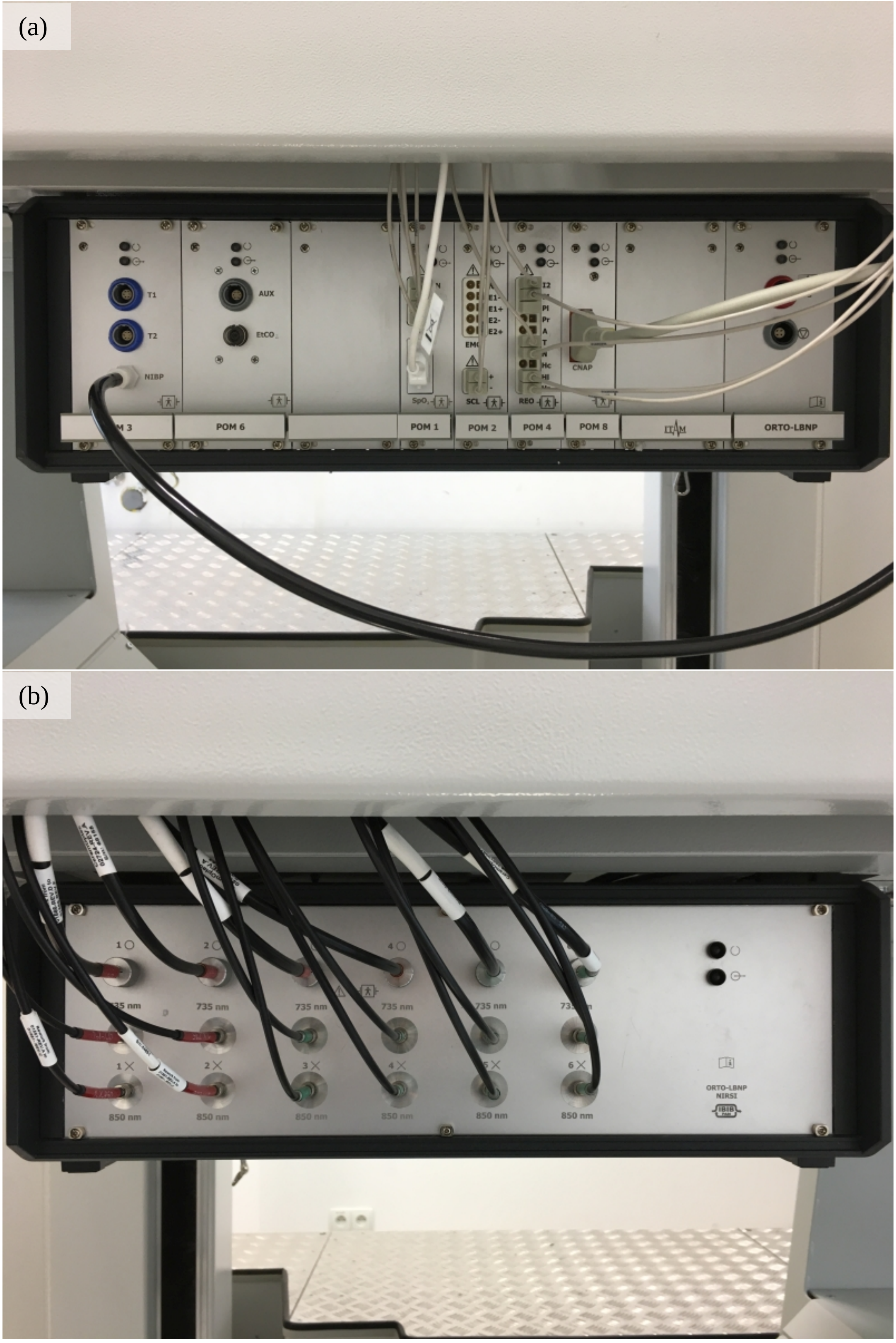

Figure S15. Medical monitoring subsystem: the (a) main module box by the *Institute of Medical Technology and Equipment*, Poland, (b) in-house-designed NIRS module box by the *Nałęcz Institute of Biocybernetics and Biomedical Engineering of the Polish Academy of Sciences*.

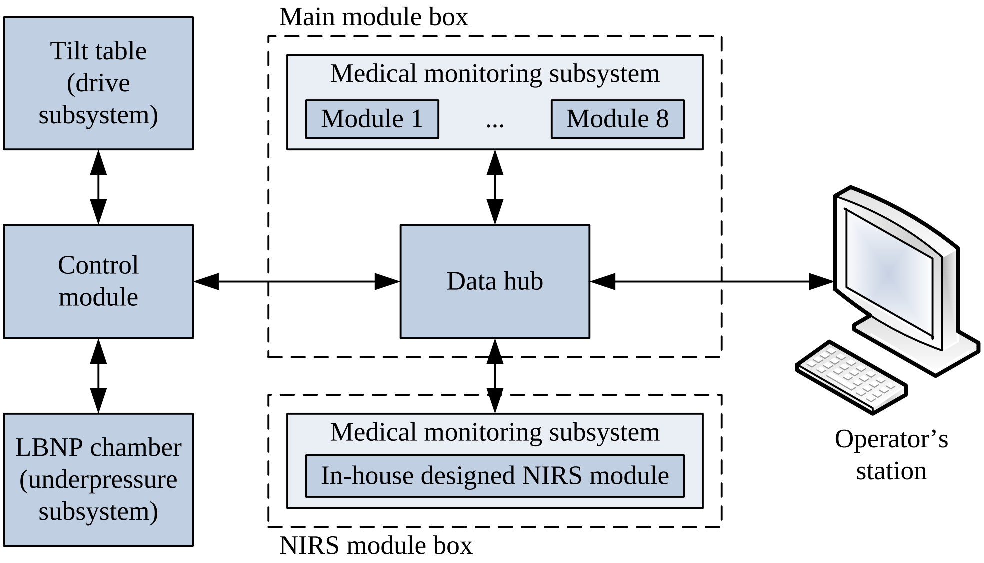

Figure S16. Block diagram of the prototype system.

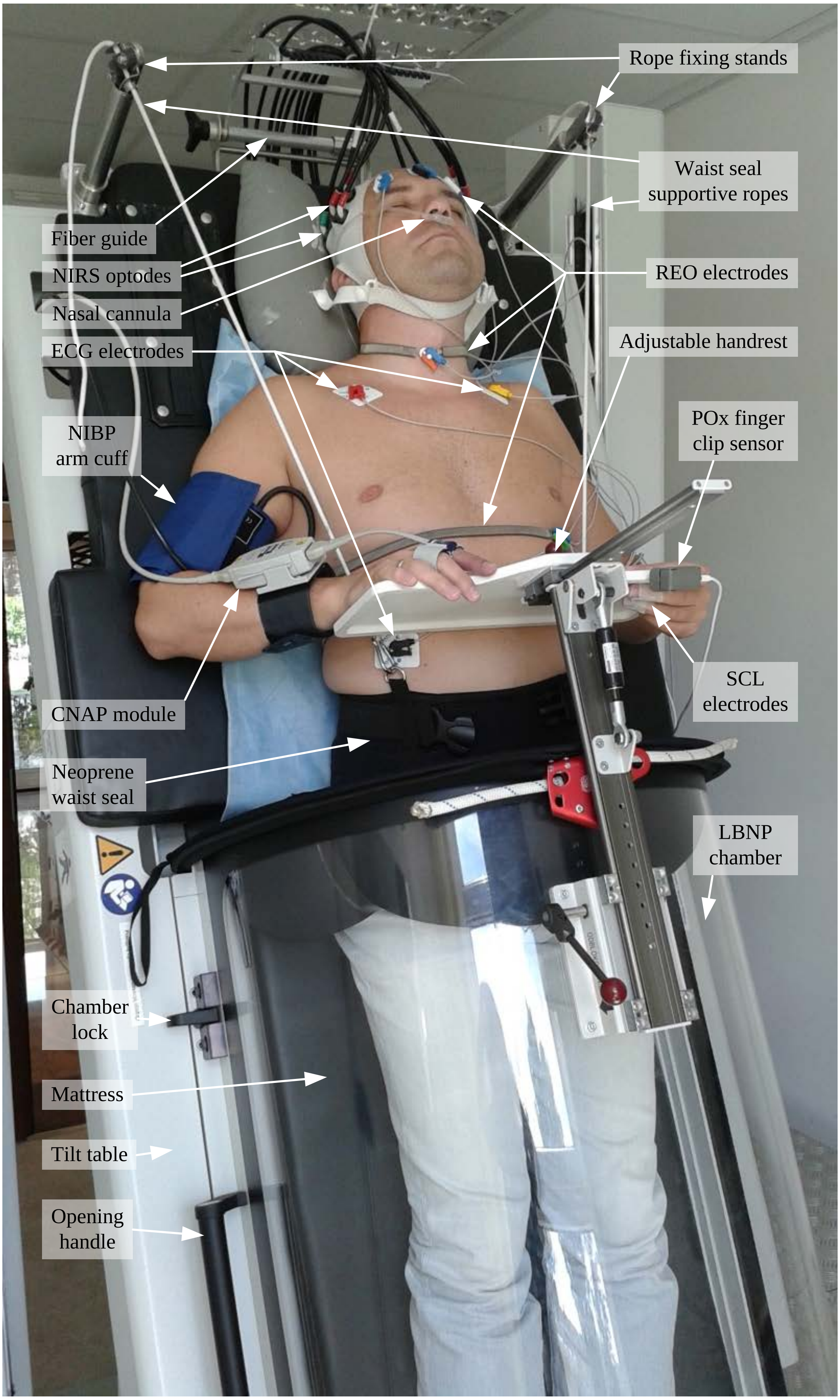

Figure S17. Subject during an examination using the prototype system.

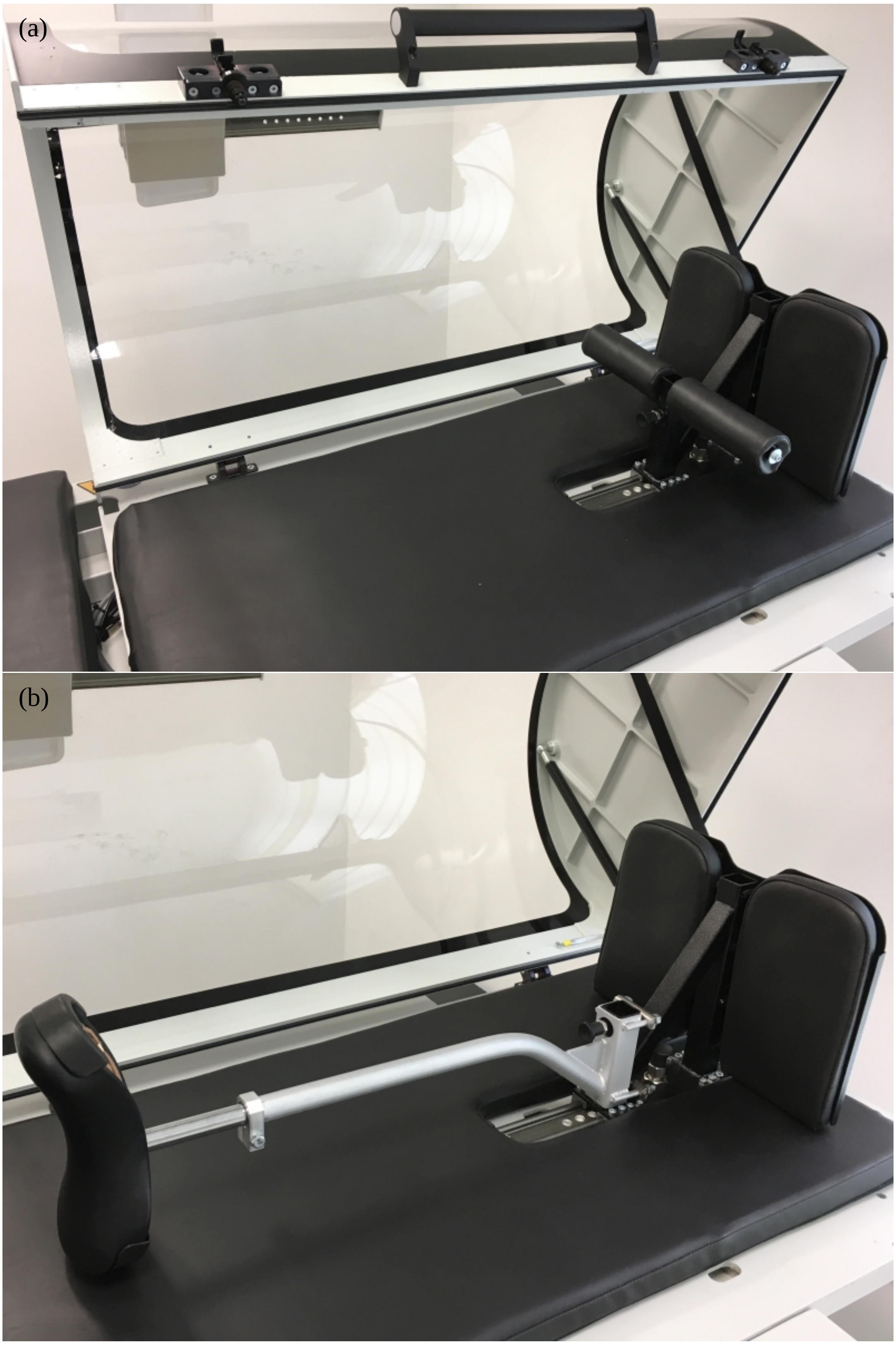

Figure S18. Photographs of the prototype system: (a) open LBNP chamber with the footboard, (b) optionally mounted saddle.

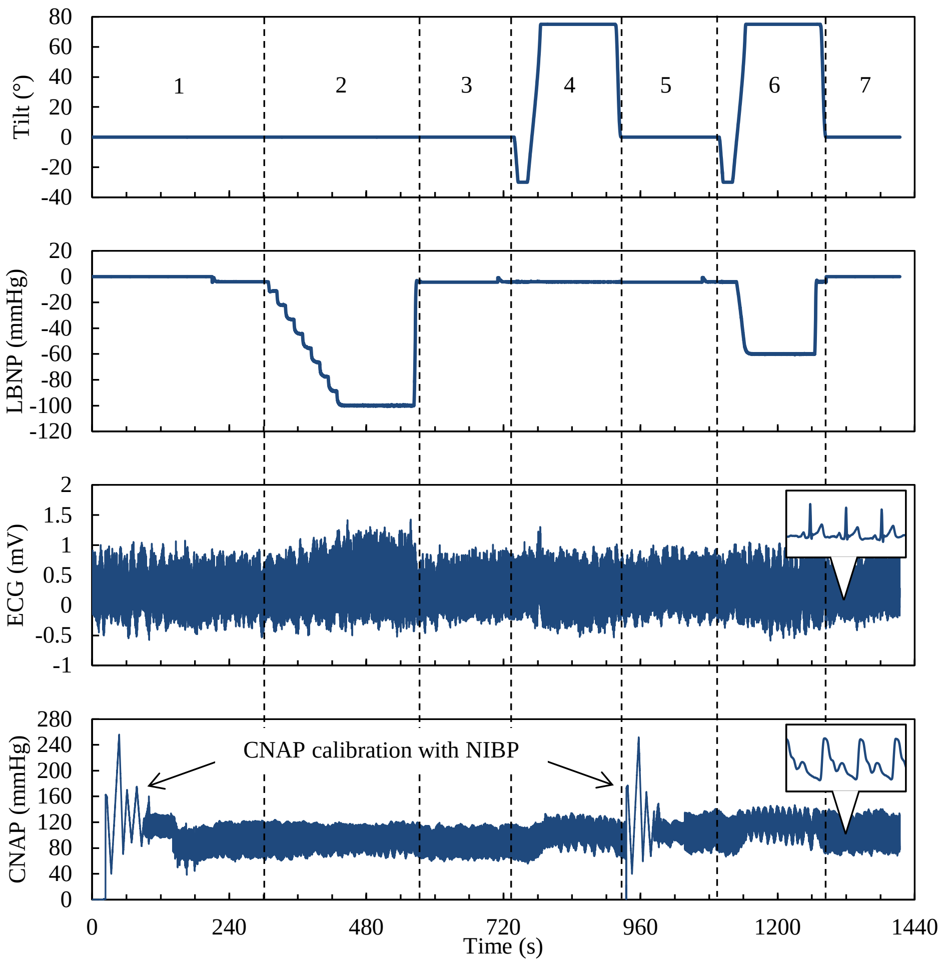

Figure S19. Example of an original recording: table tilt, LBNP profile, ECG signal, and CNAP wave.

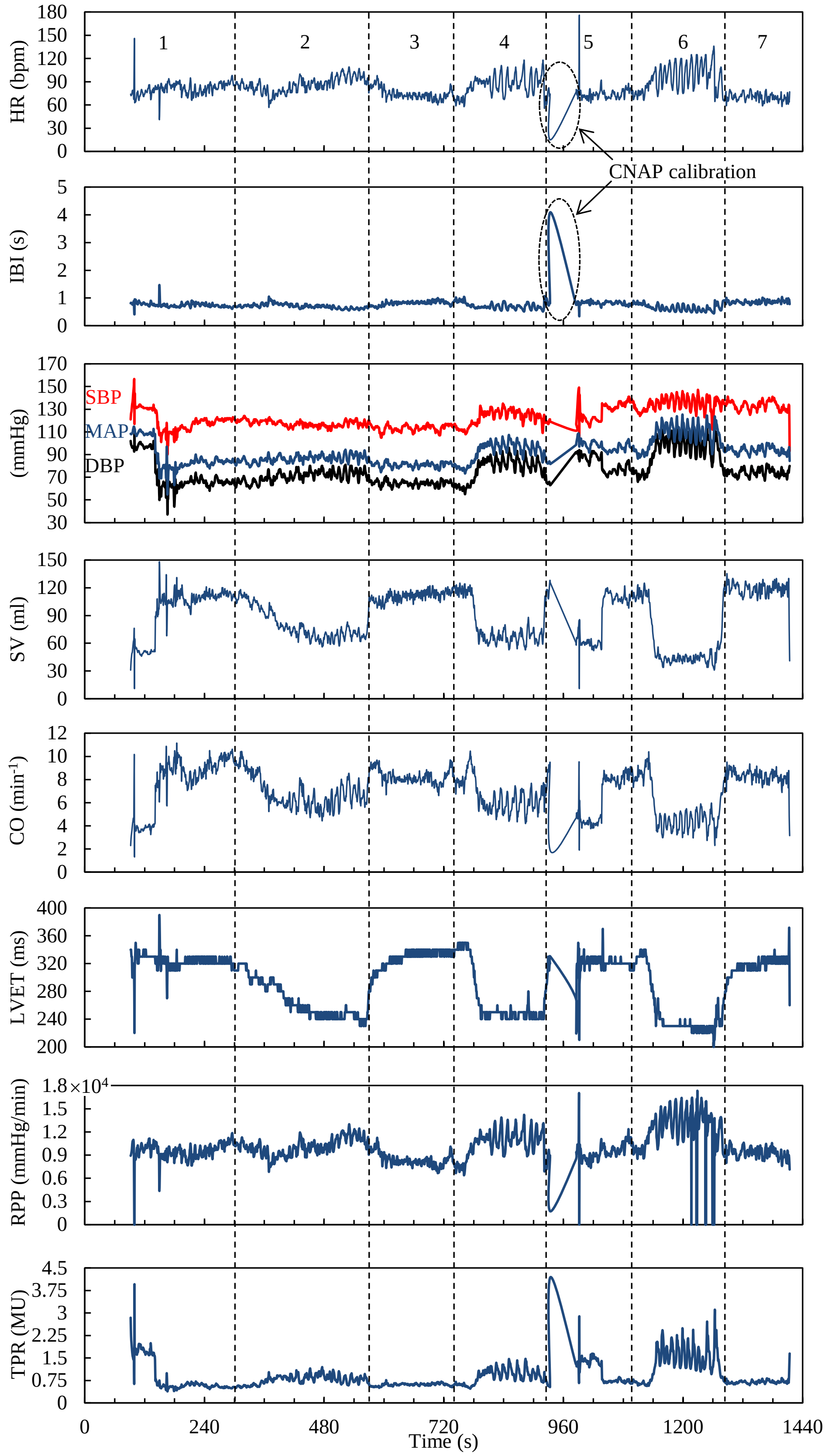

Figure S20. Traces of the physiological parameters extracted from the ECG and CNAP signals in the subsequent phases of the pilot study.

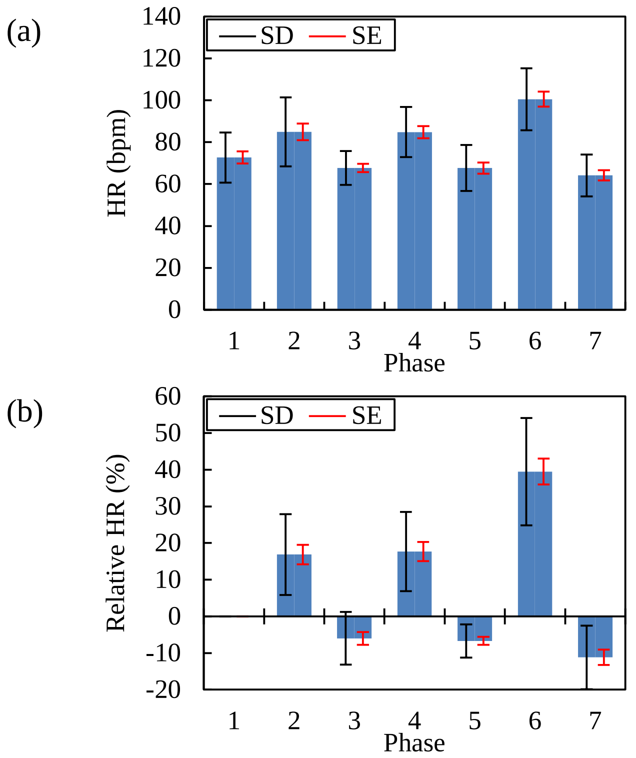

Figure S21. (a) Absolute and (b) relative (in relation to the initial level in phase 1) changes in HR in the subsequent phases of the pilot study.

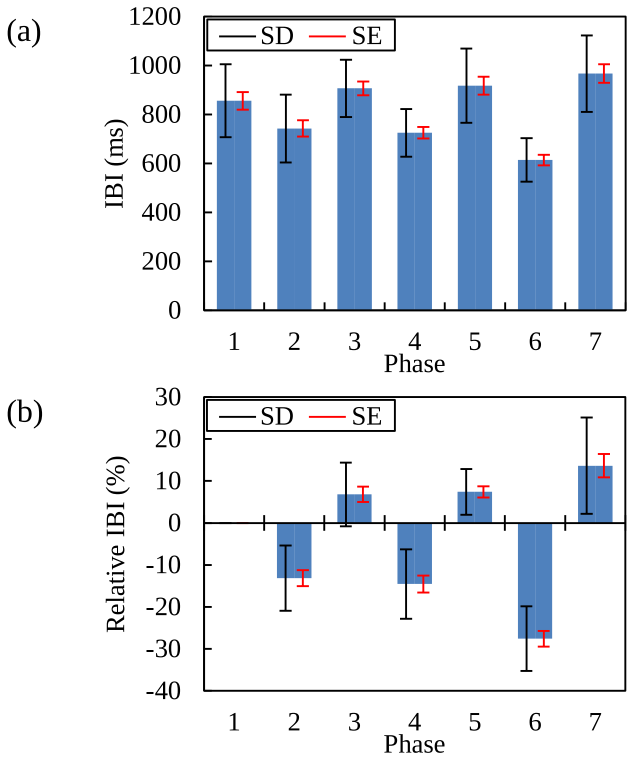

Figure S22. (a) Absolute and (b) relative changes in IBI in the subsequent phases of the pilot study.

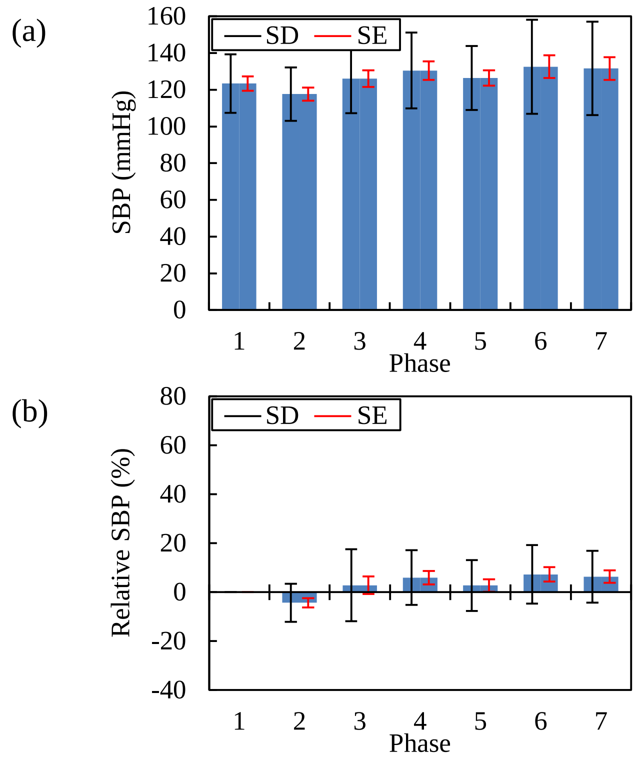

Figure S23. (a) Absolute and (b) relative changes in SBP in the subsequent phases of the pilot study.

(a)

(b)

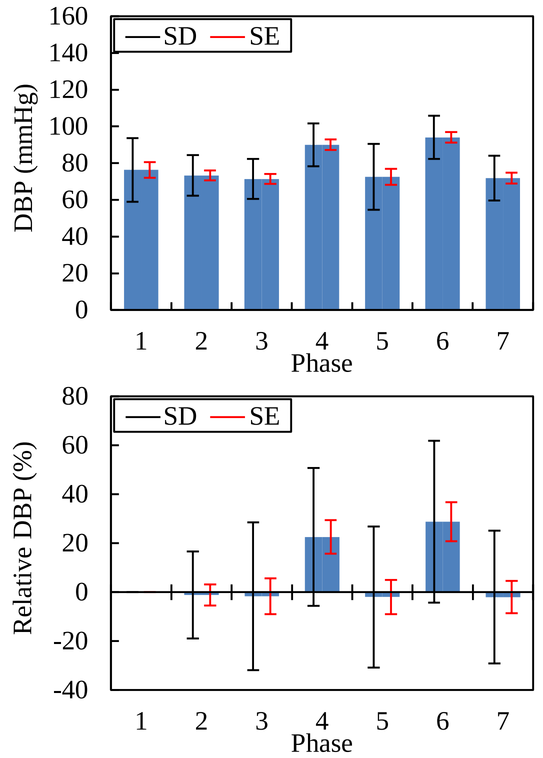

Figure S24. (a) Absolute and (b) relative changes in DBP in the subsequent phases of the pilot study.

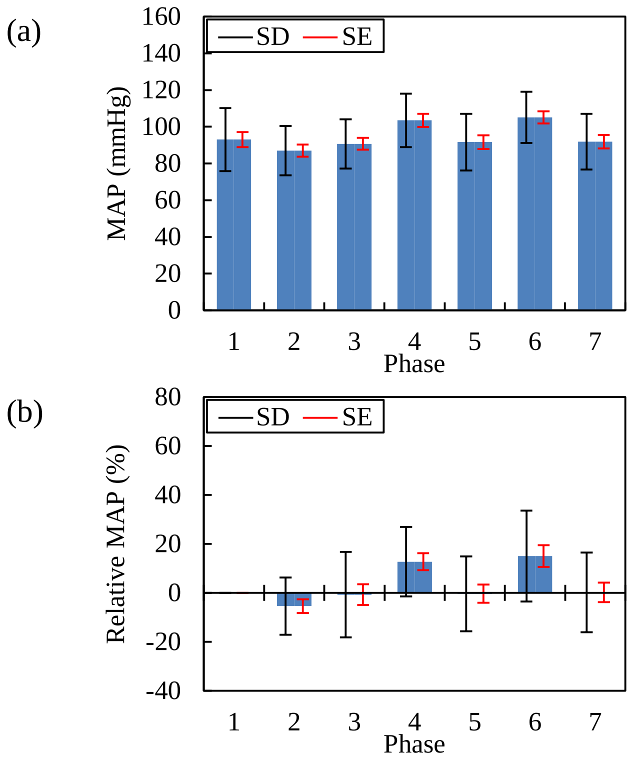

Figure S25. (a) Absolute and (b) relative changes in MAP in the subsequent phases of the pilot study.

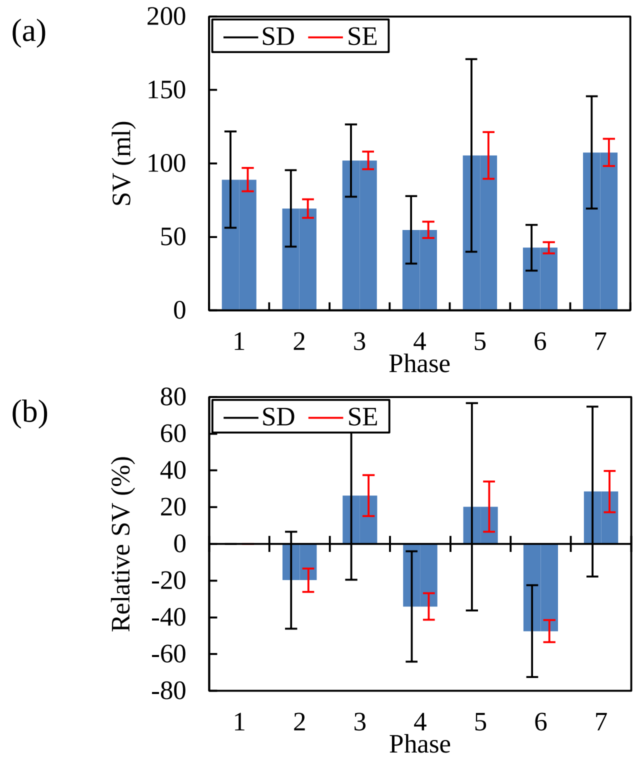

Figure S26. (a) Absolute and (b) relative changes in SV in the subsequent phases of the pilot study.

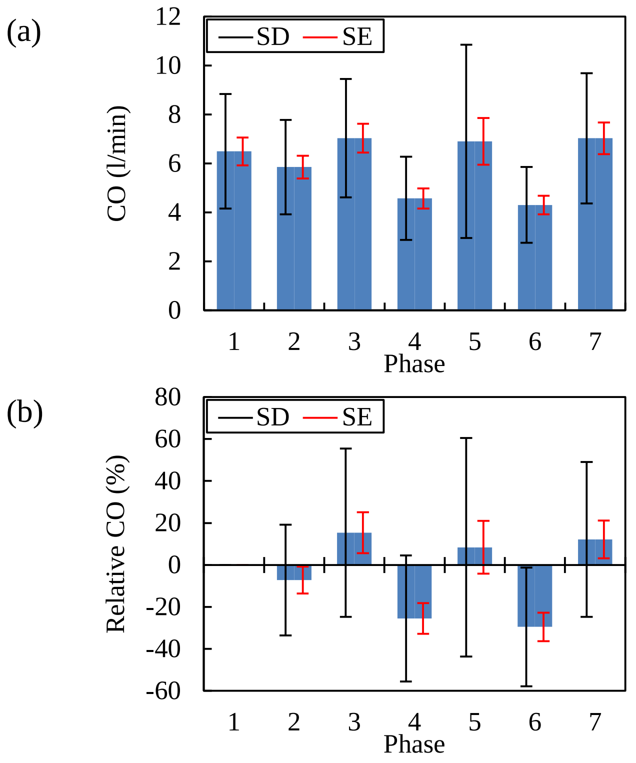

Figure S27. (a) Absolute and (b) relative changes in CO in the subsequent phases of the pilot study.

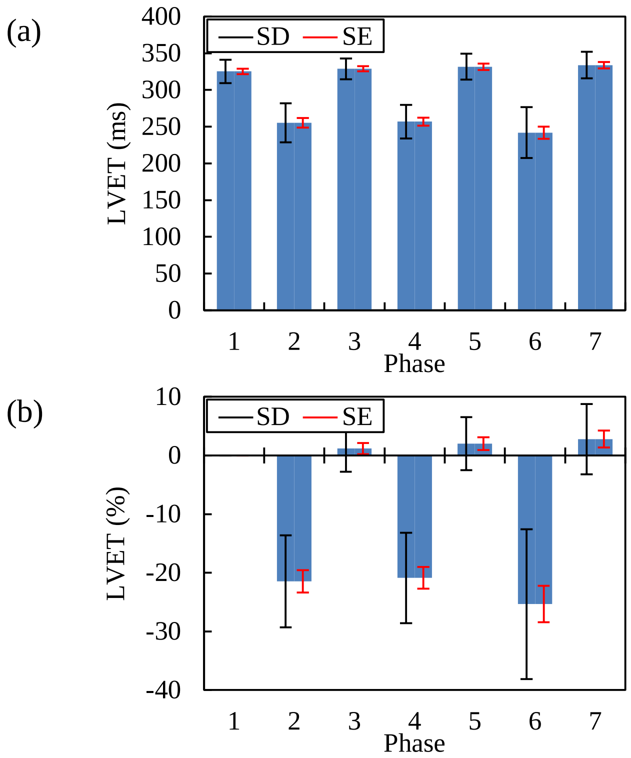

Figure S28. (a) Absolute and (b) relative changes in LVET in the subsequent phases of the pilot study.

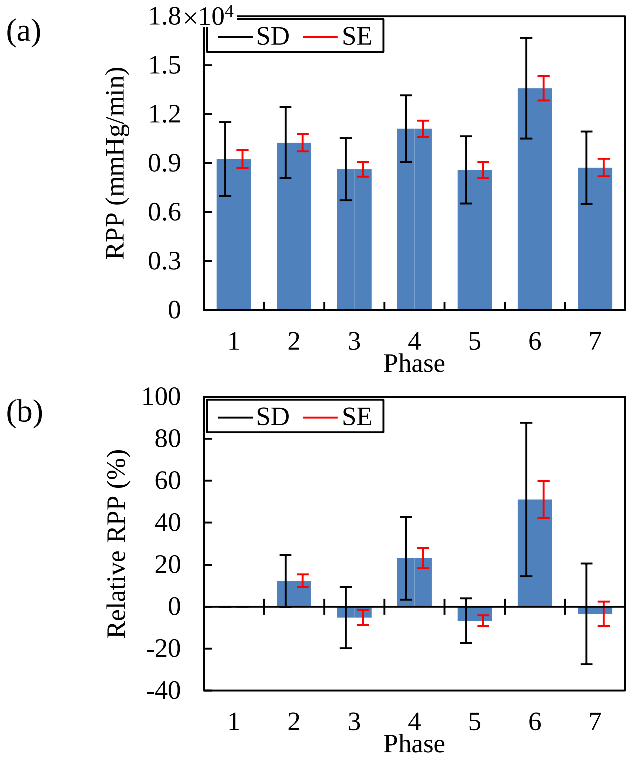

Figure S29. (a) Absolute and (b) relative changes in RPP in the subsequent phases of the pilot study.

(a)

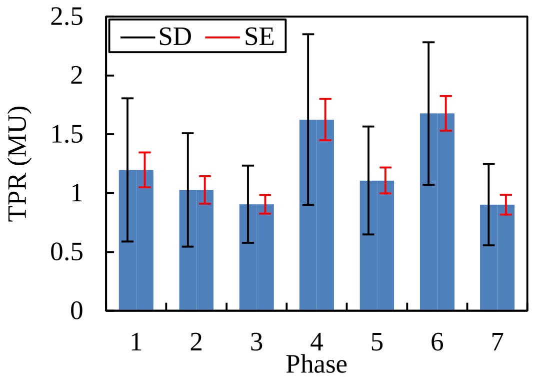

(b)

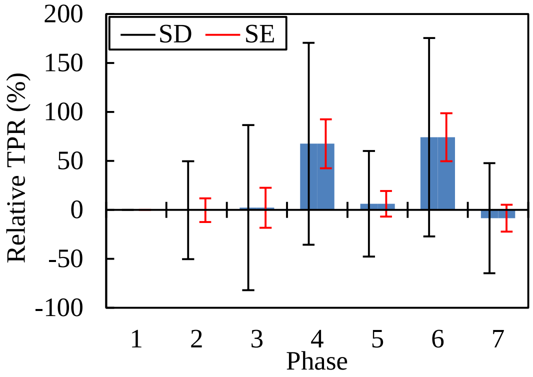

Figure S30. (a) Absolute and (b) relative changes in TPR in the subsequent phases of the pilot study.

Table S1. Results of the tilt table construction optimisation.

Criterion	Model					
	1	2	3	4	5	6
Aesthetics	4	4	4	3	5	5
Areas dangerous to patient	3	3	3	2	4	3
Areas dangerous to staff	3	3	3	2	5	4
Complexity	4	4	3	2	2	4
Cost	4	4	4	4	1	3
Degree of precision	4	3	3	3	1	3
Dimensions	4	4	4	2	2	4
Ease of cleaning	4	4	3	3	2	3
Ease of leading cables	4	4	4	4	2	3
Easy to build	5	5	5	3	1	5
Ergonomics	2	2	2	2	3	3
Rigidity, durability	1	1	1	2	4	4
Threat in case of failure	2	2	2	3	4	3
Use of commercial elements	2	2	2	2	2	4
Variable rotation axis	1	1	1	1	3	5
Weight	4	4	4	3	2	4
Points total	51	50	48	41	43	60

Rating: 1 - poor, 2 - acceptable, 3 - good, 4 - very good, 5 - excellent

Table S2. List of the mean values of the considered cardiovascular parameters in the subsequent phases of the pilot study. The significance levels for changes in the mean values obtained in the subsequent phases are shown in relation to those obtained in phase 1.

Parameter	Phase						
	1	2	3	4	5	6	7
HR (bpm)	72.65	84.90	67.69	84.81	67.65	100.49	64.12
SD	±12.01	±16.52	±8.05	±11.99	±11.02	±14.75	±10.00
Rel HR (%)	0	16.87	-5.98	17.68	-6.70	39.49	-11.19
SD	0	±11.04	±7.18	±10.78	±4.51	±14.65	±8.65
p	-	**0.000037**	**0.007633**	**0.000275**	**0.000275**	**0.000037**	**0.001616**
IBI (ms)	856.07	742.87	906.83	725.21	917.71	614.53	967.23
SD	±149.01	±138.30	±117.25	±97.43	±151.74	±88.87	±156.26
Rel IBI (%)	0	-13.15	6.81	-14.53	7.42	-27.58	13.66
SD	0	±7.80	±7.59	±8.26	±5.43	±7.70	±11.44
p	-	**0.000037**	**0.007633**	**0.000275**	**0.000275**	**0.000037**	**0.001616**
SBP (mmHg)	123.39	117.62	126.08	130.47	126.42	132.54	131.63
SD	±16.01	±14.52	±18.83	±20.68	±17.41	±25.57	±25.47
Rel SBP (%)	0	-4.30	2.85	5.96	2.76	7.31	6.37
SD	0	±7.79	±14.69	±11.18	±10.42	±12.01	±10.58
p	-	0.089555	0.808365	**0.007633**	0.808365	**0.029049**	**0.029049**
DBP (mmHg)	76.31	73.31	71.40	90.02	72.54	94.06	71.83
SD	±17.32	±11.11	±10.95	±11.71	±17.94	±11.83	±12.18
Rel DBP (%)	0	-1.11	-1.66	22.56	-1.986	28.78	-1.99
SD	0	±17.76	±30.22	±28.18	±28.76	±33.02	±27.11
p	-	0.808365	0.225253	**0.007633**	0.808365	**0.001616**	0.089555
MAP (mmHg)	93.02	87.00	90.68	103.47	91.68	105.12	91.93
SD	±17.14	±13.48	±13.41	±14.60	±15.44	±13.97	±15.17
Rel MAP (%)	0	-5.39	-0.70	12.73	-0.35	15.04	0.21
SD	0	±11.64	±17.47	±14.19	±15.29	±18.52	±16.27
p	-	0.089555	0.808365	**0.001616**	0.808365	**0.029049**	0.808365
SV (ml)	88.98	69.36	102.04	54.79	105.38	42.65	107.49
SD	±32.77	±26.04	±24.60	±22.90	±65.52	±15.61	±38.31
Rel SV (%)	0	-19.75	26.37	-34.09	20.23	-47.50	28.51
SD	0	±26.43	±45.90	±30.00	±56.43	±25.05	±46.22
p	-	**0.029049**	0.225253	**0.007633**	0.466854	**0.000275**	**0.029049**
CO (l/min)	6.50	5.85	7.03	4.58	6.90	4.31	7.03
SD	±2.34	±1.93	±2.42	±1.70	±3.95	±1.55	±2.66
Rel CO (%)	0	-7.16	15.36	-25.49	8.44	-29.52	12.15
SD	0	±26.36	±40.13	±30.00	±52.04	±28.27	±36.89
p	-	**0.029049**	0.466854	**0.029049**	0.225253	**0.007633**	0.225253
LVET (ms)	325.25	255.28	328.76	256.89	331.55	241.8	333.84
SD	±15.84	±26.47	±14.21	±22.77	±17.66	±34.65	±17.97
Rel LVET (%)	0	-21.45	1.18	-20.87	2.01	-25.35	2.79
SD	0	±7.85	±3.96	±7.70	±4.51	±12.79	±5.99
p	-	**0.000037**	0.466854	**0.000037**	0.089555	**0.000275**	0.225253
RPP (mmHg/min)	0.92×10^4	1.03×10^4	0.86×10^4	1.11×10^4	0.86×10^4	1.36×10^4	0.87×10^4
SD	$\pm0.23\times10^4$	$\pm0.22\times10^4$	$\pm0.19\times10^4$	$\pm0.20\times10^4$	$\pm0.21\times10^4$	$\pm0.31\times10^4$	$\pm0.22\times10^4$
Rel RPP (%)	0	12.28×10^4	-5.23×10^4	23.07×10^4	-6.70×10^4	51.05×10^4	-3.43×10^4
SD	0	$\pm12.44\times10^4$	$\pm14.66\times10^4$	$\pm19.73\times10^4$	$\pm10.59\times10^4$	$\pm36.55\times10^4$	$\pm24.02\times10^4$
p	-	**0.007633**	0.089555	**0.000275**	**0.007633**	**0.000037**	0.089555
TPR (MU)	1.20	1.03	0.91	1.62	1.11	1.68	0.90
SD	±0.61	±0.48	±0.33	±0.73	±0.46	±0.61	±0.35
Rel TPR (%)	0	-0.31	2.25	67.55	6.14	74.28	-8.46
SD	0	±49.98	±84.36	±103.12	±53.95	±101.34	±56.28
p	-	0.466854	0.466854	**0.029049**	0.808365	0.089555	**0.029049**

Rel - relative values calculated with reference to the initial level in phase 1
MU - medical units
Values in bold are statistically significant ($p \leq 0.05$)

Table S3. List of the most important features of the tilt tables with combined LBNP chambers shown in the literature and the main results of research works carried out with them.

Features			Subjects; experimental procedure; main outcome	Reference
**Tilt table**	**LBNP chamber**	**Biomedical monitoring**		
Manually adjustable in the range from -15° to +60° with 10° increment, steel frame, adjustable right-armrest, adjustable footplate	Manually adjustable down to -60 mmHg, high power vacuum cleaner, plastic and wooden elements, neoprene waist seal	ECG, blood pressure, brachial and cerebral blood flow, end-tidal tensions of oxygen ($P_{ET}O_2$) and carbon dioxide ($P_{ET}CO_2$)	No information on number of subjects; 20-minute supine rest, 20-minute 60° HUT at 30-second transition to upright, 3 sequences of 10-minute HUTs and LBNP at -20, -40 and -60 mmHg, respectively, recovery; suggestion that tilt testing with combined LBNP can be considered as a "gold standard"	Protheroe et al.
Manually adjustable in the range from -90° to +90°, steel frame	Manually adjustable down to -50 mmHg, industrial 6-horsepower vacuum, wooden box, kayak spray skirt	ECG, HR, blood pressure, MAP, SV, CO, $P_{ET}CO_2$, transcranial Doppler, cerebral blood velocity (CBV)	13 subjects (males); 5-minute supine rest, 10-minute -50 mmHg supine LBNP, 15-minute recovery, 10-minute 45° HDT or -45° HUT and -50 mmHg LBNP, recovery; body position has no effect on CBV responses during LBNP, CBV decreases during LBNP	Tymko et al.
Manually adjustable in the range from 0° to -15°, steel frame	Manually adjustable down to -25 mmHg, industrial vacuum pump, steel and Plexiglas elements, neoprene seal	Photoplethysmography, tibial and skin blood flows, NIRS	11 subjects (8 males and 3 females); 5-minute sitting on the tilt table, 5-minute supine rest, 5-minute -15° HDT, 10-minute -15° HDT and -25 mmHg LBNP; short-term LBNP can reduce the HDT-induced increase in microvascular blood flow in the tibia to values consistent with sitting posture	Siamwala et al.
Automatically adjustable in the range from -70° to +70°, table attached to the movable steel rim, adjustable footplate	Automatically adjustable down to -60 mmHg by an advanced subsystem for generating underpressure, steel and Plexiglas elements, neoprene seal	ECG, HR, blood pressure, MAP, SBP, DBP, impedance cardiography, SV	15 subjects (males); 30-minute supine rest, 5-minute 70° HUT followed by additional 20 mmHg LBNP that is increased by additional 10 mmHg LBNP every three minutes until presyncope occurred; mean standing time from begin HUT to presyncope was determined	Grasser et al.
Automatically adjustable in the range from -45° to +80° with up to 45°/s rate of tilt changes, steel frame, adjustable handrest, adjustable footboard	Automatically adjustable down to -100 mmHg with up to 20 mmHg/s rate of underpressure changes, Al and Plexiglas elements, neoprene waist seal	ECG, HR, IBI, blood pressure, MAP, SBP, DBP, SV, CO, LVET, RPP, TPR, pulse oximetry, EMG, SCL, body temperature, impedance reography, EEG, respiration curve, NIRS	17 subjects (14 males and 3 females); 5-minute supine rest, -100 mmHg supine LBNP, 75° HUT, -60 mmHg LBNP associated with 75° HUT, separated by 3-minute resting phases; recovery; the prototype system can map any pre-programed tilt and LBNP profiles, confirmation of the efficiency of performing experimental procedures	Dziuda et al.

Author contributions

Ł.D. prepared figures S7, S8, S14-S20 and supplementary data files; M.Ś. prepared figures S21-S30; M.S. prepared figures S1-S6 and S9-S12; P.K. prepared figure S13.
